# Which Exercise Interventions Can Most Effectively Improve Reactive Balance in Older Adults? A Systematic Review and Network Meta-Analysis

**DOI:** 10.3389/fnagi.2021.764826

**Published:** 2022-01-18

**Authors:** Youngwook Kim, Michael N. Vakula, David A. E. Bolton, Christopher J. Dakin, Brennan J. Thompson, Timothy A. Slocum, Masaru Teramoto, Eadric Bressel

**Affiliations:** ^1^Department of Kinesiology and Health Science, Utah State University, Logan, UT, United States; ^2^Department of Special Education and Rehabilitation Counseling, Utah State University, Logan, UT, United States; ^3^Division of Physical Medicine & Rehabilitation, University of Utah, Salt Lake City, UT, United States

**Keywords:** older adults, aging, balance, reactive balance, exercise, falls, accidental falls, fall prevention

## Abstract

**Background:**

Reactive balance is the last line of defense to prevent a fall when the body loses stability, and beneficial effects of various exercise-based interventions on reactive balance in older adults have been reported. However, their pooled evidence on the relative effects has yet to be described.

**Objective:**

To review and evaluate the comparative effectiveness of various exercise-based interventions on reactive balance in older adults.

**Methods:**

Nine electronic databases and reference lists were searched from inception to August 2021. Eligibility criteria according to PICOS criteria were as follows: (1) population: older adults with the mean age of 65 years or above; (2) intervention and comparison: at least two distinct exercise interventions or one exercise intervention with a no-exercise controlled intervention (NE) compared in each trial; (3) outcome: at least one measure of reactive balance; (4) study: randomized controlled trial. The main network meta-analysis was performed on data from the entire older adult population, involving all clinical conditions as well as healthy older adults. Subgroup analyses stratified by characteristics of participants (healthy only) and reactive balance outcomes (simulated slip or trip while walking, simulated forward falls, being pushed or pulled, and movable platform) were also conducted.

**Results:**

Thirty-nine RCTs (*n* = 1388) investigating 17 different types of exercise interventions were included in the network meta-analysis. Reactive balance training as a single intervention presented the highest probability (surface under the cumulative ranking (SUCRA) score) of being the best intervention for improving reactive balance and the greatest relative effects vs. NE in the entire sample involving all clinical conditions [SUCRA = 0.9; mean difference (95% Credible Interval): 2.7 (1.0 to 4.3)]. The results were not affected by characteristics of participants (i.e., healthy older adults only) or reactive balance outcomes.

**Summary/Conclusion:**

The findings from the NMA suggest that a task-specific reactive balance exercise could be the optimal intervention for improving reactive balance in older adults, and power training can be considered as a secondary training exercise.

## Introduction

The World Health Organization reported that approximately 28–35% of people aged 65 or above experience at least one fall each year, and the frequency of falls increases with age and frailty level (World Health Organization, 2008). Among various intrinsic risk factors for falls, gait and balance problems have been considered the strongest risk factors (Deandrea et al., 2010; Ambrose et al., 2013). Balance can be mechanistically achieved and maintained by a complex set of sensorimotor control systems including the multisensory (visual, somatosensory, and vestibular system) integration into the central nervous system and the subsequent motor output of the musculoskeletal system (Shumway-Cook and Woollacott, 2017). However, older adults show age-related decline in sensorimotor systems, which in turn increases the risks of falls (Mahoney et al., 2019; Osoba et al., 2019). Given the inherent and inevitable age-related degeneration in sensorimotor systems, it is becoming increasingly clear that in order to prevent potential repercussions, such as aging-related disease, disabilities, injuries, and falls, there is an urgent need for effective interventions to decelerate or even reverse the retrogression in the balance and gait control systems (Kim et al., 2020; Sibley et al., 2021).

In daily life, reactive balance, referred to as the ability to control balance in response to mechanical disturbances, plays a critical role in avoiding and adapting to the complex environments that menace postural stability. The WHO Global Report on Falls Prevention in Older Age reported that factors related to the physical environment, for instance, uneven sidewalks, unmarked obstacles, and slippery surfaces, are some of the most common causes (30–50%) of falls in older adults (World Health Organization, 2008). Notably, slips and trips were the most prevalent causes of falls in regards to circumstances in older adults (Berg et al., 1997). Reactive balance strategies, such as swaying around the ankle or hip joints, taking a reactive step, or reaching to grasp a handhold (Shumway-Cook and Woollacott, 2017), need to be executed promptly so as to avoid falls following a postural perturbation. In the same vein, the balance recovery reactions have also shown age-related differences in older adults vs. young adults and in fallers vs. non-fallers (Alissa et al., 2020; Okubo et al., 2021).

There is a considerable amount of literature on the effects of a variety of interventions on reactive balance, including several systematic reviews and meta-analyses focusing on older adults (Bohm et al., 2015; Lesinski et al., 2015; McCrum et al., 2017; Moore et al., 2019). However, there remain some limitations in the prior syntheses. First, the exercise interventions were limited to only those interventions focused on balance or strength training despite the existence of many studies that use exercises that specifically train reactive balance. Consequently, to the best of our knowledge, none of the previous reviews or meta-analyses have considered comparative effects between different types of exercises and the efficacy of multifaceted exercise interventions with more than one type of exercise on reactive balance. Thus, there is a need for a more comprehensive and inclusive analysis utilizing precise coding of exercise types targeting specific biological systems and functional aspects for better prescriptive guidance (Sibley et al., 2021). Second, the systematic review by Moore et al. (2019) who examined the effectiveness of active physical training interventions on reactive balance did not perform a quantitative synthesis (Moore et al., 2019). Consequently, there remains a lack of pooled evidence on the relative effects of different exercise interventions on reactive balance. Moreover, a conventional pairwise meta-analysis is restricted to a head-to-head comparison of only two different interventions, and thus, RCTs with other types of exercise interventions, that are also effective, can potentially be excluded. To tackle this problem, a network meta-analysis (NMA) is well suited, because it facilitates comparisons of multiple pairs of interventions in one statistical model (Dias et al., 2018).

Therefore, the current study aimed to quantitatively synthesize the available evidence of RCTs in detail using a systematic review and NMA to: (1) combine information from all available randomized comparisons of a set of exercise interventions for reactive balance in older adults; (2) to appraise the relative effects of different exercise interventions on reactive balance; and (3) to determine the ranking of each to provide practical and clinical suggestions to design evidence-based exercise programs for reactive balance. The research question was as follows: “What type of exercise intervention is most effective in improving overall measures as well as each measure of reactive balance in older adults?”.

## Methods

The protocol was prospectively registered in the PROSPERO database (CRD42021256638) and conducted in accordance with the PRISMA extension statement for network meta-analysis (Hutton et al., 2015).

### Eligibility Criteria

The population of interest included older adults with a sample mean age of 65 years or above with no restriction on the injury or disorder type and with no history of falls studied in various research settings (e.g., community, clinics, and long-term care facilities). Studies were included, if at least two experimental groups participated in each of the different exercise interventions or if there was at least one exercise intervention group with a no-exercise control group. Studies involving any non-exercise interventions (e.g., medication, electrical stimulation, or nutritional supplement) were excluded. Details regarding the exercise interventions must have been provided in the reports. The studies must have included at least one reactive balance assessment, which is defined in this study as an assessment using mechanical postural perturbation during a static or dynamic steady-state task. The studies included in this review were restricted to randomized controlled trials (RCTs) and written in the English language.

### Search Strategy

The following electronic databases were initially searched by one reviewer (Y.K.) from the inception to February 2021: MEDLINE, EBSCO, CINAHL, SPORTDiscus, PsycINFO, PubMed. WorldCat.org, OpenGrey.eu, and PROQUEST were additionally searched for unpublished trials. To keep this search up to date, an updated search followed in August 2021 by two reviewers (YK and MV). Earlier reviews and bibliographies of included studies were reviewed for additional potentially relevant trials. The combination of the following keywords was employed for the database searches: (aged OR aging OR old^*^ OR elder^*^ OR senior^*^) AND (exercise OR train^*^ OR activit^*^ OR rehabilitat^*^ OR therap^*^ OR physiotherapy OR hydrotherapy OR conditioning OR exertion OR recreation^*^ OR aerobic^*^ OR stretch^*^ OR strengthen^*^ OR walk^*^ OR jog^*^ OR run^*^ OR cycl^*^ OR pilates OR yoga OR tai chi OR ai chi OR dance OR swim^*^) AND (reactive postural response OR stepping response OR perturbation OR slip perturbation OR reactive balance OR reactive stepping OR protective stepping OR compensatory stepping OR anticipatory postural adjustment^*^ OR compensatory postural adjustment^*^ OR anticipatory postural response^*^ OR compensatory postural response^*^ OR anticipatory adjustment^*^ OR compensatory adjustment^*^ OR postural adaptation^*^ OR postural stabili^*^ation OR automatic postural response^*^ OR postural stepping response^*^) AND (random^*^).

### Study Selection

After exporting the references and removing duplicates, titles and abstracts of records were screened independently by two reviewers (YK and MV) according to the eligibility criteria. Full texts of all potentially relevant trials were subsequently retrieved and reviewed to confirm the final eligible trials. Any disagreements were resolved via consensus, and when any disagreement was elusive, a third reviewer (EB) acted as an arbiter.

### Data Extraction and Coding

A total of 46 eligible studies were reviewed and coded in REDCap (https://www.projectredcap.org/) by one reviewer (YK) and confirmed by a second reviewer (M.V.). Any disagreements were resolved via consultation with a third reviewer (EB). The extracted data included: (1) study characteristics; (2) baseline demographics of participants; (3) exercise interventions; (4) reactive balance outcome measures; and (5) results. Exercise categorizations developed by Howe et al. and Sibley et al. were modified in consideration of the purpose of the current research and applied to the coding (Table 1) (Howe et al., 2011; Sibley et al., 2021).

**Table 1:** Definitions of exercise types.

**Exercise type**	**Code**	**Definitions**
Single balance exercise including reactive balance component	SBR	An intervention including a balance exercise with one or more mechanical postural perturbations given during the exercise
Single balance exercise not including reactive balance component	SBNR	An intervention including a balance exercise without any mechanical postural perturbations
Multiple balance exercises including reactive balance component	MBR	An intervention including more than one type of balance exercise with one or more mechanical postural perturbations given during one of the exercises
Multiple balance exercises not including reactive balance component	MBNR	An intervention including more than one type of balance exercise without any mechanical postural perturbations
Unspecified balance exercise	balUS	Balance exercise without any details given in the original article
Gait training including reactive balance component	gaitR	An intervention including gait training with one or more mechanical postural perturbations given during the exercise
Gait training not including reactive balance component	gaitNR	An intervention including gait training without any mechanical postural perturbations
Whole body vibration	WBV	Any activity performed on a machine with a vibrating platform
Strength	str	Exercise that uses the external resistance load (e.g., body weight, resistance bands, machines) to force skeletal muscles contract.
Power	pw	Exercise that applies the maximum amount of force (muscle contraction against a resistance) in the shortest period of time.
3D exercise	3d	Exercise that requires multi-dimensional movements with a specific name of the exercise (e.g., Yoga, dance, Tai Chi)
Flexibility	flex	Exercise that intends to restore or maintain the optimal range of motion (ROM) available to a joint or joints.
Functional training	FT	Exercise that utilizes functional activities as the training stimulus that is based on the theoretical concept of task specificity
Aerobic	aer	Exercise aimed at cardiovascular conditioning. It is aerobic in nature and simultaneously increases the heart rate and the return of blood to the heart.
No exercise	NE	A group received none of the exercise interventions listed above

Means (M) and standard deviations (SD) for all eligible outcomes of reactive balance measures at baseline and post-intervention were extracted for the analysis. Missing data related to eligibility and study outcomes (i.e., data not reported either in a text or on publicly accessible data repositories) were requested to the corresponding authors via email. In the case of no response after one month, a second request was sent, if another month elapsed without response, the data were considered irretrievable. If the requested, but not retrieved data were presented in a graphical format rather than numeric data (e.g., tabular format), Engauge Digitizer 12.1 (http://markummitchell.github.io/engauge-digitizer/) was applied for data digitization and extraction.

### Risk of Bias

To ascertain an overall and study-level risk of bias for each trial, a pair of reviewers (YK and MV) independently determined the bias arising from the following domains using the Cochrane risk of bias tool (RoB 2): (1) randomization process; (2) deviations from the intended interventions; (3) missing outcome data; (4) measurement of the outcome; and (5) selection of the reported result (Sterne et al., 2019). Each domain was assigned a judgement of “low risk,” “some concerns,” or “high risk.” Disagreements were resolved through discussion or referral to a third reviewer (EB).

### Data Synthesis and Statistical Analysis

Considering indeterminate baseline similarities of reactive balance measures in several studies, change values from baseline to post-intervention were calculated or directly extracted from the published data. If there were more than one post-intervention measure (e.g., post-intervention and follow-up), only the data immediately following the termination of the intervention phase was used. SDs for changes from baseline (pre) to post-intervention (post) were calculated using the following formula (Higgins et al., 2019):


$$\begin{array}{l} SD_{change}=\sqrt{{SD_{pre}}^{2}+{SD_{post}}^{2}-2*Corr*SD_{pre}*SD_{post}} \end{array}$$

Corr in the SD_change_ equation is the correlation coefficient describing how similar the pre and post-interventions were across participants. When the correlation coefficient was not reported, it was set as 0.5 (Fu et al., 2008; Bruderer-Hofstetter et al., 2018; Lai et al., 2018; Wu et al., 2021). In the case of a lower score signifying better performance in reactive balance measures (e.g., reaction time), scale directions were adjusted by multiplying −1 to the M_change_ data, which led to a greater effect size indicating an improvement. Missing SDs were imputed from standard errors (SE), 90 or 95% confidence intervals (CI). Using the M_change_ and SD_chang_ data, standardized mean differences (SMD) and standard errors (SE) were calculated.

To include multi-arm trials, two approaches were adopted to avoid a unit-of-analysis error (Rücker et al., 2017; Higgins et al., 2019). First, all relevant experimental intervention groups composed of the same categories of exercises were combined into a single group. This step enabled a single pairwise comparison between a combined group and a comparison group in each study. Second, in the case of heterogeneous exercise types across all intervention groups, we included all relevant comparisons as a series of two-arm comparisons and reflect the fact that comparisons within multi-arm studies are correlated (Schwarzer et al., 2015). Accordingly, adjusted SEs of the two-arm comparisons in each multi-arm study were computed using “netmeta” package in R software. The majority of the eligible trials consisted of multiple outcomes in each trial. When multiple SMDs were estimated in a single study, therefore, a pooled SMD with SE was computed.

To estimate the comparative effectiveness of exercise-based interventions on reactive balance, we implemented NMA, which incorporates both direct (i.e., head-to-head comparison from pairwise meta-analysis) and indirect comparisons (i.e., from network meta-analysis) in one statistical model. A Bayesian framework of NMA was conducted using Markov chain Monte Carlo simulations, and non-informative prior distributions for treatment effects were adopted (Lunn et al., 2000; Dias et al., 2018). A random-effects model was used considering the clinical and methodological between-study heterogeneity (Sutton et al., 2000; Borenstein et al., 2009). The NMA was conducted for all available exercise interventions included in at least two trials. The analyses utilized a burn-in period (50,000 iterations) and a follow-up period (100,000 iterations) to minimize bias of initial values when the chain reached its target distribution (Brooks and Gelman, 1998). The convergence was assessed using the trace plot, density plot, and Brooks-Gelman-Rubin diagnostic statistics (Brooks and Gelman, 1998).

The overall geometry of the network was presented in a network graph. Based on Bayesian posterior rank probabilities, the ranking of exercise interventions was estimated using a hierarchical tool, the surface under the cumulative ranking curve (SUCRA) score, measured on a scale from 0 (theoretically the worst) to 1 (the best). In addition, a network forest plot was produced with the “no exercise (NE)” as a reference intervention. The posterior distribution of the SMDs was reported using the mean differences fto the reference intervention with 95% credible intervals (CrI), which indicate that there is a 95% probability that the unobserved (unknown) effect estimates would fall within the intervals (Hespanhol et al., 2019). If a 95% CrI contains zero (i.e., null effect representing the null hypothesis), the effect can be considered statistically insignificant (Hespanhol et al., 2019). The relative effects with 95% CrI of all pairs of exercise interventions were reported in a matrix. Consistency, which is the most important assumption underlying a NMA and indicates agreement between direct and indirect estimates in the network (Salanti et al., 2014), was checked using the node-splitting analysis. The original intention of the first subgroup analysis was to conduct a network meta-analysis stratified by characteristics of participants (i.e., healthy and disease-specific). However, due to the insufficient number of exercise interventions to establish a network in each disease category (e.g., Parkinson's disease), the first subgroup analysis was performed by the inclusion of studies with healthy older adults only (78% of all studies) who did not have any disease, injury, or disability at the time of the studies. The second subgroup analysis was conducted by grouping the outcome measures by the types of reactive balance outcomes: (1) simulated slip or trip while walking; (2) simulated forward falls; (3) being pushed or pulled; (4) movable platform; and (5) balance test battery. A sensitivity analysis was carried out using a frequentist framework NMA to appraise the robustness of the results. Sources of statistical heterogeneity and small study bias were not explored due to the insufficient number of trials (k ≤ 5) for each comparison. All data syntheses and statistical analyses were conducted using “Gemtc” (version 1.0-1), “rjags” (version 4-10), and “netmeta” (version 1.4-0) packages in R (Version 4.1.0, R Foundation for Statistical Computing, Vienna, Austria).

## Results

### Study Selection

**Figure 1:**
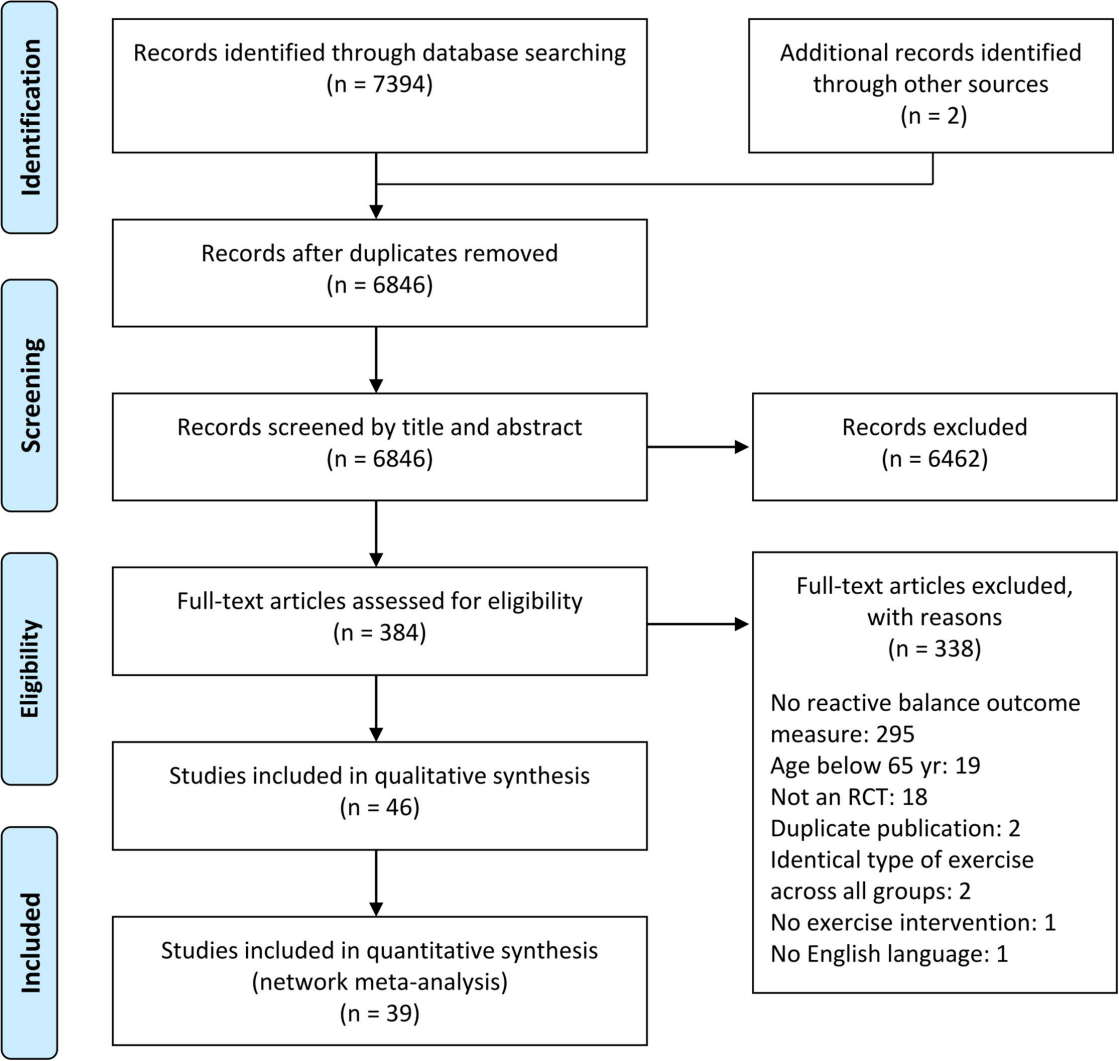
PRISMA flow diagram of study selection.

A total of 7,394 records were retrieved from electronic databases and two additional records were obtained from other sources, of which 384 studies remained after removing duplicates and screening titles and abstracts. Based on the full-text screening, 46 records fulfilled the eligibility criteria and thus were included for qualitative analysis (i.e., systematic review), whereas seven studies were additionally excluded from the quantitative analysis (i.e., network meta-analysis) due to data not being reported and not irretrievable (Kim and Lockhart, 2010; Okubo et al., 2019; Wang et al., 2019), exercise types not included in the network (Allin et al., 2020; Cabrera-Martos et al., 2020), exercise intervention included in only one trial (Lacroix et al., 2016), and no continuous data reported (Beling and Roller, 2009), resulting in a total of 39 studies for NMA. The schematic flow chart for the selection process is presented in Figure 1, and all included studies are listed in Supplementary Table 1.

### Characteristics of Included Studies

The eligible studies represented a total of 1,745 older adults, included in both pre and post-intervention analyses, with the mean age of 71.9 ± 3.9 years (ranged from 65.3–80.9 years). The majority of the studies exclusively included community-dwelling healthy older adults (*k* = 36). Ten studies reported on older adults selected for a specific disease or medical condition, such as Parkinson's disease (*k* = 6), post-surgical interventions for knees, hips, or backs (*k* = 2), postmenopausal women with osteopenia (*k* = 1), and chronic stroke (*k* = 1).

The duration and frequency of the exercise interventions ranged from 1 week to 1 year, 1–5 sessions/week, and 15–90 min/session. Of the 46 studies, 16 executed multicomponent (i.e., multifaceted) exercise interventions in at least one group. Reactive balance was assessed before and after the exercise interventions by use of laboratory-induced slip, trip, and falls, external impacts (e.g., pulling or pushing a body part), platform translation, and treadmill perturbation (e.g., rapid change of the speed) while participants were performing a steady-state task, such as standing or walking. Twenty studies provided training with a postural perturbation while standing or walking, and 11 of which implemented task-specific training (i.e., comparable reactive balance task included in the assessment and training) (Wolf et al., 1997; Bieryla et al., 2007; Beling and Roller, 2009; Mansfield et al., 2010; Parijat and Lockhart, 2012; Jagdhane et al., 2016; Morat et al., 2019; Okubo et al., 2019; Wang et al., 2019; Arghavani et al., 2020; Rieger et al., 2020). The characteristics of the participants and exercise interventions are presented in Tables 2, 3, respectively. Outcome measures and main findings are summarized in Supplementary Table 2.

**Table 2:** Characteristics of participants.

**Study**	**Disease category**	**Sample size (post-intervention)**	**Attrition rate (%)**	**Age (years)**
Allin et al. (2020)	Healthy	34 (29)	15	70.4
Arampatzis et al. (2011)	Healthy	55 (38)	31	67.7
Arghavani et al. (2020)	Healthy (fallers: 6 months)	60 (49)	18	69.6
Beling and Roller (2009)	Healthy	23 (19)	17	80.0
Bieryla et al. (2007)	Healthy	12 (11)	8	73.3
Bogaerts et al. (2007)	Healthy	220 (161)	27	67.1
Cabrera-Martos et al. (2020)	Parkinson's	44 (44)	0	76.5
Cherup et al. (2019)	Parkinson's	42 (35)	17	71.2
Chyu et al. (2010)	Postmenopausal women with osteopenia	61 (53)	13	71.9
Donath et al. (2016)	Healthy	59 (48)	19	69.7
Gatts and Woollacott (2007)	Healthy (balance deficiency without any neurological disorder); Arthritis, back, knee, or hip surgery not excluded.	22 (19)	14	77.6
Gatts (2008)	Healthy (balance deficiency without any neurological disorder); Arthritis, back, knee, or hip surgery not excluded.	22 (19)	14	77.6
Granacher et al. (2006)	Healthy	60 (60)	0	66.5
Granacher et al. (2009)	Healthy	40 (40)	0	67.0
Hamed et al. (2018)	Healthy	63 (47)	25	71.2
Hatzitaki et al. (2009)	Healthy	56 (56)	0	70.9
Hu and Woollacott (1994)	Healthy	24 (24)	0	75.2
Inacio et al. (2018)	Healthy	18 (18)	0	71.9
Jagdhane et al. (2016)	Healthy	6 (6)	0	73.3
Kim and Lockhart (2010)	Healthy	18 (18)	0	NS
Klamroth et al. (2019)	Parkinson's	43 (37)	14	65.3
Lacroix et al. (2016)	Healthy	66 (60)	9	72.8
Li et al. (2009)	Healthy	50 (40)	20	65.3
Ma et al. (2019)	Healthy	33 (24)	27	69.8
Mansfield et al. (2010)	Healthy (fallers: 5 years)	34 (30)	12	69.7
Marigold et al. (2005)	chronic stroke	59 (48)	19	67.8
Morat et al. (2019)	Healthy	51 (45)	12	69.4
Ni et al. (2014)	Healthy	48 (39)	19	74.2
Ochi et al. (2015)	Healthy	20 (20)	0	80.6
Okubo et al. (2019)	Healthy	44 (41)	7	72.1
Pamukoff et al. (2014)	Healthy (some lower extremity mobility dysfunction)	20 (15)	25	70.8
Parijat and Lockhart (2012)	Healthy	24 (24)	0	72.7
Parijat et al. (2015a)	Healthy	24 (24)	0	72.4
Parijat et al. (2015b)	Healthy	24 (24)	0	72.4
Pluchino et al. (2012)	Healthy	40 (27)	33	72.1
Qutubuddin et al. (2007)	Parkinson's	22 (15)	32	72.8
Rieger et al. (2020)	Healthy	30 (30)	0	71.0
Rossi et al. (2014)	Healthy	46 (46)	0	67.5
Santos et al. (2017)	Parkinson's	40 (40)	0	67.8
Schlenstedt et al. (2015)	Parkinson's	40 (32)	20	75.7
Shimada et al. (2003)	Healthy	34 (32)	6	80.9
Sohn and Kim (2015)	Healthy	18 (18)	0	73.7
Thomas and Kalicinski (2016)	Healthy	24 (24)	0	67.1
Wang et al. (2019)	Healthy	146 (146)	0	72.7
Wolf et al. (1997)	Healthy	72 (54)	25	76.9
Wooten et al. (2018)	Healthy (fallers: 1 year)	30 (16)	47	72.6

**Table 3:** Summary of exercise interventions.

**Study**	**Dosage**		**Total duration (week)**	**Exercise interventions**		
	**Min/session**	**Time/week**		**Group1**	**Group2**	**Group3**
Allin et al. (2020)	30-60	2	2	SBR + gaitR	MBNR + gaitNR + str	
Arampatzis et al. (2011)	90	2	14	MBR	SBNR + str	NE
Arghavani et al. (2020)	60	3	8	SBR	MBNR + gaitNR + str	NE
Beling and Roller (2009)	60	3	12	MBR + gaitNR + flex + str	NE	
Bieryla et al. (2007)	15	1	1	gaitR	gaitNR	
Bogaerts et al. (2007)	40-90	3	1 year	MBNR + WBV	SBNR + str + flex + aer	NE
Cabrera-Martos et al. (2020)	45	3	8	FT	FT + flex	
Cherup et al. (2019)	60	2	12	pw	Str	
Chyu et al. (2010)	60	3	24	3d	NE	
Donath et al. (2016)	66	2	8	3d	MBNR	NE
Gatts and Woollacott (2007)	90	5	3	3d	SBNR + flex	
Gatts (2008)	90	5	3	3d	SBNR + flex	
Granacher et al. (2006)	60	3	13	str	SBNR	NE
Granacher et al. (2009)	60	3	13	str	NE	
Hamed et al. (2018)	90	2	14	str	SBR	NE
Hatzitaki et al. (2009)	30	3	4	SBNR	SBNR	NE
Hu and Woollacott (1994)	60	10 sessions (total)	15 days (total)	SBNR	NE	
Inacio et al. (2018)	15	3	8	pw	str	
Jagdhane et al. (2016)	60	3	4	SBR	NE	
Kim and Lockhart (2010)	NR	NR	8	str	MBNR	NE
Klamroth et al. (2019)	40	2	8	gaitR	gaitNR	
Lacroix et al. (2016)	45	3	12	MBNR + str + pw	MBNR + str + pw	NE
Li et al. (2009)	60	4 for 6weeks, 7 for 10 weeks	16	3d	NE	
Ma et al. (2019)	60	2	12	3d	NE	
Mansfield et al. (2010)	30	3	6	SBR	SBNR + flex	
Marigold et al. (2005)	60	3	10	MBR + gaitNR	SBNR + flex	
Morat et al. (2019)	40	3	8	SBR	SBNR	NE
Ni et al. (2014)	60	2	12	3d	MBNR	3d
Ochi et al. (2015)	30	3	12	MBNR + WBV	SBNR + str	
Okubo et al. (2019)	40	3	1	gaitR	gaitNR	
Pamukoff et al. (2014)	60	3	6	pw	str	
Parijat and Lockhart (2012)	40	1	1	gaitR	gaitNR	
Parijat et al. (2015a)	35–55	1	1	gaitR	gaitNR	
Parijat et al. (2015b)	35–55	1	1	gaitR	gaitNR	
Pluchino et al. (2012)	60	2	8	MBNR + gaitNR	3d	MBNR
Qutubuddin et al. (2007)	30	2	4	balUS	MBNR + gaitNR	
Rieger et al. (2020)	NS	1	1	gaitR	gaitNR	
Rossi et al. (2014)	40	3	6	SBNR	NE	
Santos et al. (2017)	60	2	8	str + flex	MBR + gaitNR	
Schlenstedt et al. (2015)	60	2	7	str	MBR	
Shimada et al. (2003)	40	2–3	12	MBNR	gaitNR	str + flex
Sohn and Kim (2015)	60	3	8	str	balUS	NE
Thomas and Kalicinski (2016)	70	2	6	MBNR	NE	
Wang et al. (2019)	30	1	1	gaitR	gaitNR	
Wolf et al. (1997)	60	1–2	15	MBR	NE	3d
Wooten et al. (2018)	45	3	6	MBNR	3d	

*SBR, Single balance exercise including reactive balance component; SBNR, Single balance exercise not including reactive balance component; MBR, Multiple balance exercises including reactive balance component; MBNR, Multiple balance exercises not including reactive balance component; balUS, Unspecified balance exercise; gaitR, Gait training including reactive balance component; gaitNR, Gait training not including reactive balance component; WBV, Whole body vibration; str, Strength; pw, Power; 3d, 3D exercise; FT, Functional training; flex, Flexibility; aer, Aerobic; NE, No exercise*.

### Risk of Bias

**Figure 2:**
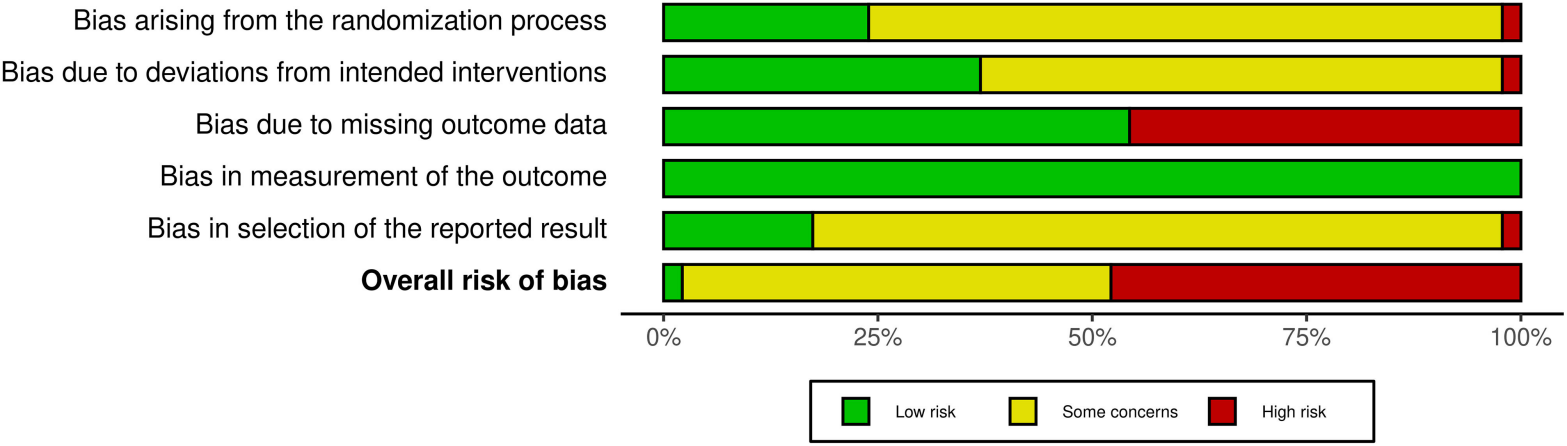
Summary of the distributions of the reviewers' judgements across the studies for each risk of bias domain.

The summary of the risk of bias assessment across all included studies is presented in Figure 2. Detailed results of the assessment are reported in Supplementary Table 3. Overall, the majority of outcomes were at some concerns (50%) and high risk (48%), and only one study was rated as at low risk. Missing outcome data (46%) was the most influential source of high risk of bias. Selection of the reported result (83%), randomization process (76%), and deviations from intended interventions (61%) were also common sources of bias.

### Network Meta-Analysis

**Figure 3:**
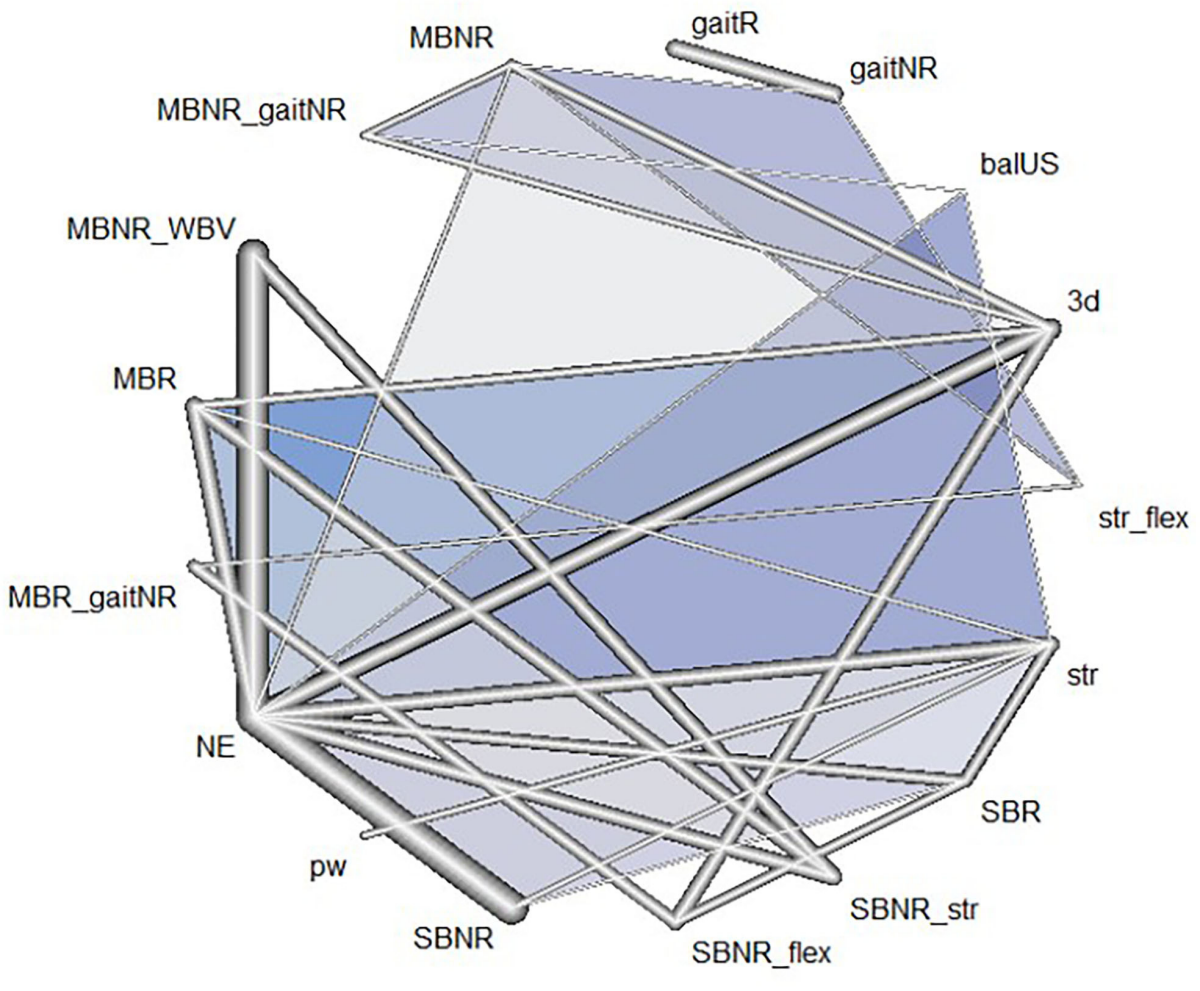
Network geometry of the included exercise programs: Each line indicates a direct comparison of two different exercise programs. The thickness of the edge is proportional to the number of direct comparisons in the network. Different exercise types combined in one program are connected via underscores. The blue triangles refer to multi-arm trials comprised of three exercise programs in the nodes. SBR, Single balance exercise including reactive balance component; SBNR, Single balance exercise not including reactive balance component; MBR, Multiple balance exercises including reactive balance component; MBNR, Multiple balance exercises not including reactive balance component; balUS, Unspecified balance exercise; gaitR, Gait training including reactive balance component; gaitNR, Gait training not including reactive balance component; WBV, Whole body vibration; str, Strength; pw, Power; 3d, 3D exercise; flex, Flexibility; aer, Aerobic; NE, No exercise.

Data from a total of 39 studies (*n* = 1388, age = 71.5 ± 3.9 years) were included in the NMA. Of the 15 exercise types reported in Table 1, 14 types were included in the NMA as functional training was implemented in only one study and consequently included in a disconnected network (Cabrera-Martos et al., 2020). There were 11 multi-arm trials, and three of which consisted of two groups sharing the same exercise type and the third group with another type (Hatzitaki et al., 2009; Ni et al., 2014; Lacroix et al., 2016); thus, data in these two groups were combined into a single group. Two exercise groups in studies by Gatts and Woollacott (2007) and Gatts (2008), str and NE groups in studies by Granacher et al. (2006, 2009), and two exercise groups in studies by Parijat et al. (2015a,b) shared the same participants, respectively. Thus, each of the aforementioned pairs of studies was combined as a single study in NMA. Overall, 17 exercise interventions with either single or multiple exercise components were included in the NMA. The geometric distribution of the network is depicted in Figure 3. When a study involves a trial arm with a combination of the pre-categorized exercise types, the combination was considered as another distinct exercise intervention.

**Figure 4:**
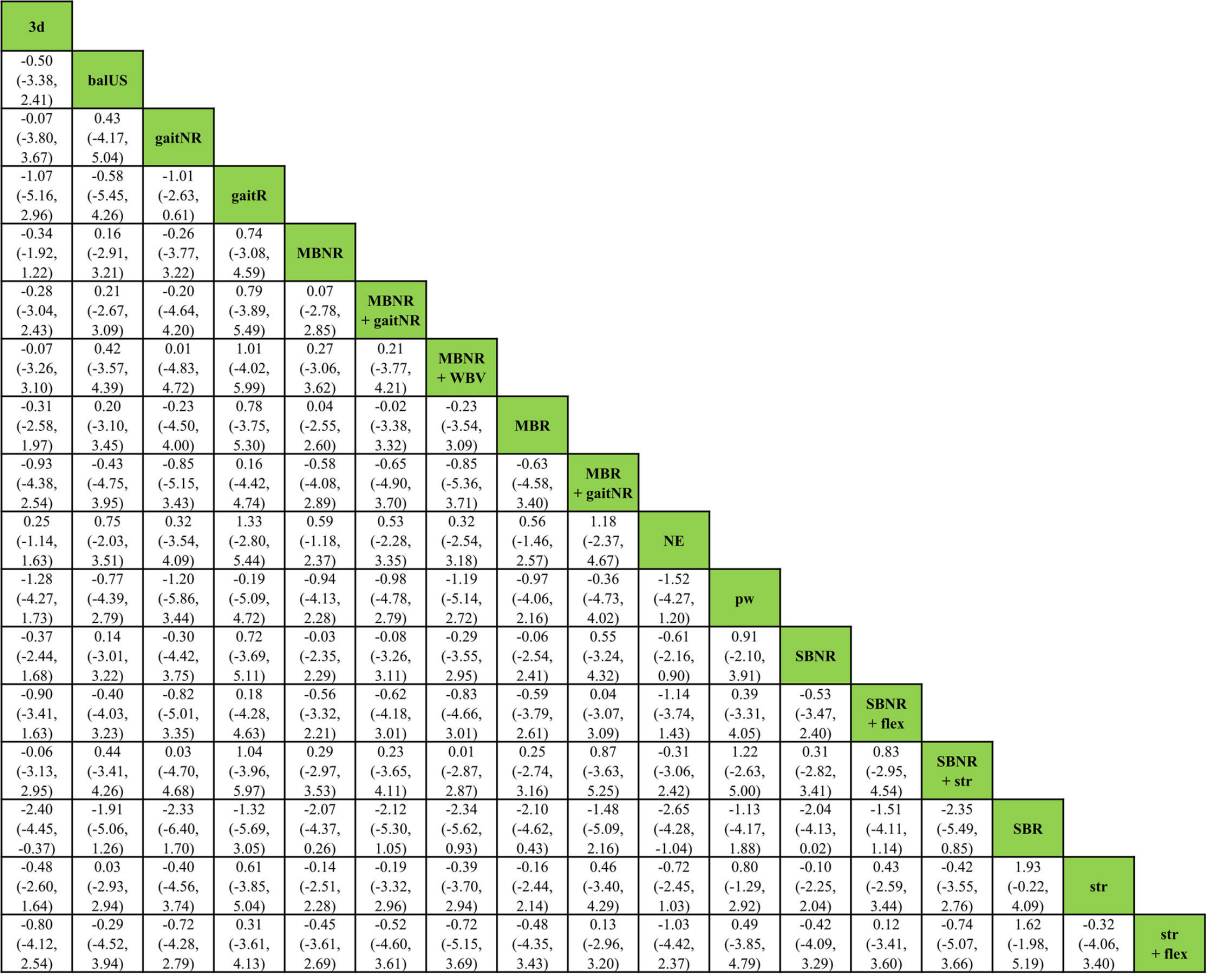
Relative effect estimates with 95% credible intervals of all pairs of exercise interventions.

Estimates of all exercise programs against all others in NMA were reported in a matrix (Figure 4). In the 17 exercise programs, SBR displayed the highest probability of being the most effective exercise intervention (SUCRA score = 0.90) for improving reactive balance, followed by pw (SUCRA score = 0.67) and gaitR (SUCRA score = 0.62) (Table 4).

**Table 4:** Ranking of exercise interventions.

**Bayesian framework**			**Frequentist framework**		
**Ranking**	**Exercise**	**SUCRA score**	**Ranking**	**Exercise**	**P-score**
1	SBR	0.90	1	SBR	0.94
2	pw	0.67	2	pw	0.70
3	gaitR	0.62	3	gaitR	0.64
4	SBNR + flex	0.58	4	SBNR + flex	0.61
5	MBR + gaitNR	0.58	5	MBR + gaitNR	0.60
6	str + flex	0.55	6	str + flex	0.57
7	balUS	0.49	7	balUS	0.49
8	str	0.49	8	str	0.49
9	SBNR	0.46	9	SBNR	0.46
10	MBNR	0.46	10	MBNR	0.45
11	MBR	0.45	11	MBR	0.44
12	MBNR + gaitNR	0.44	12	MBNR + gaitNR	0.43
13	MBNR + WBV	0.40	13	MBNR + WBV	0.38
14	SBNR + str	0.40	14	SBNR + str	0.37
15	gaitNR	0.39	15	gaitNR	0.37
16	3d	0.35	16	3d	0.33
17	NE	0.27	17	NE	0.23

*SBR, Single balance exercise including reactive balance component; SBNR, Single balance exercise not including reactive balance component; MBR, Multiple balance exercises including reactive balance component; MBNR, Multiple balance exercises not including reactive balance component; balUS, Unspecified balance exercise; gaitR, Gait training including reactive balance component; gaitNR, Gait training not including reactive balance component; WBV, Whole body vibration; str, Strength; pw, Power; 3d, 3D exercise; flex, Flexibility; aer, Aerobic; NE, No exercise*.

**Figure 5:**
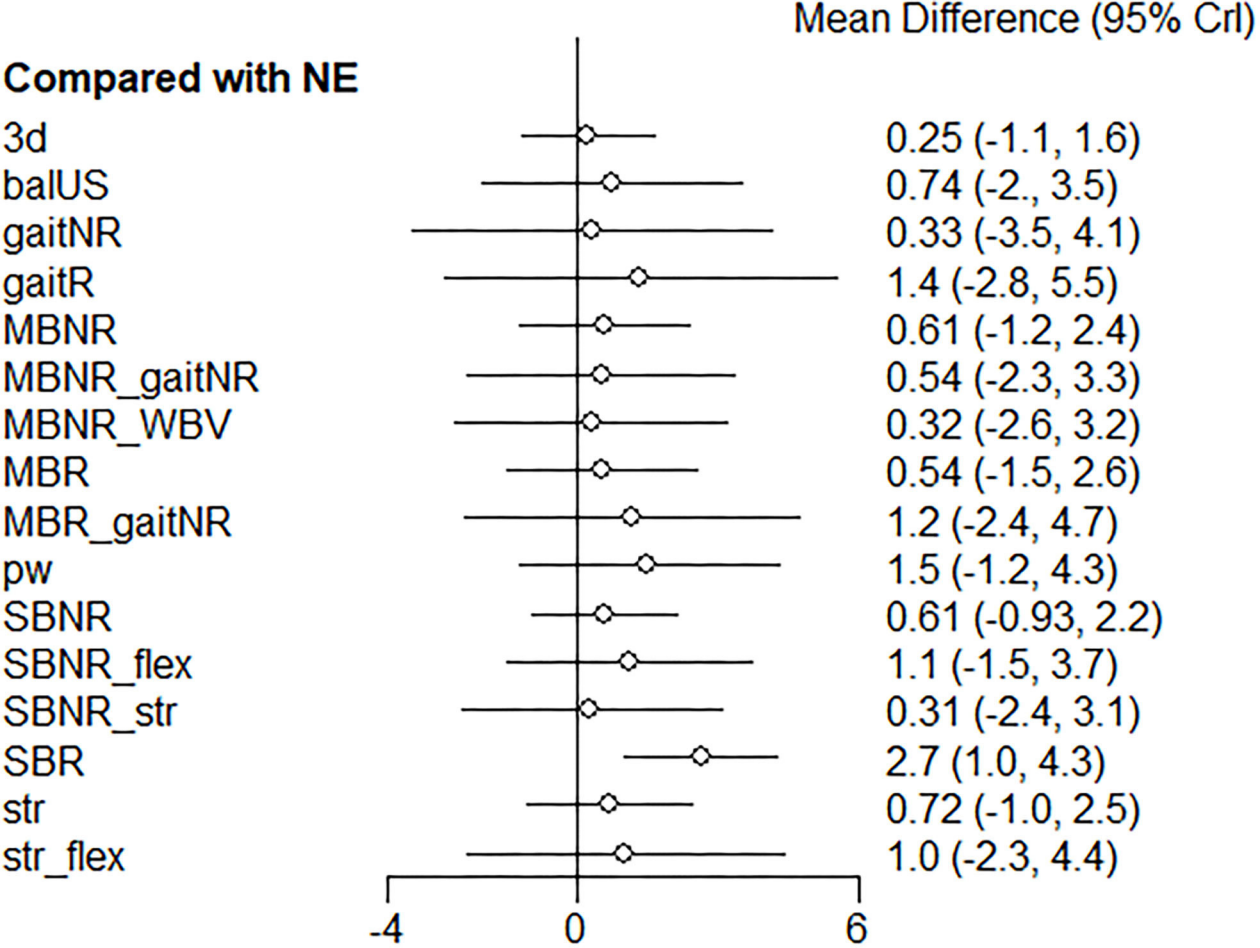
Forest plot of the relative effects of exercise interventions with a no-exercise as a reference group. SBR, Single balance exercise including reactive balance component; SBNR, Single balance exercise not including reactive balance component; MBR, Multiple balance exercises including reactive balance component; MBNR, Multiple balance exercises not including reactive balance component; balUS, Unspecified balance exercise; gaitR, Gait training including reactive balance component; gaitNR, Gait training not including reactive balance component; WBV, Whole body vibration; str, Strength; pw, Power; 3d, 3D exercise; flex, Flexibility; aer, Aerobic; NE, No exercise.

The relative treatment effect estimates of each exercise program with the no-exercise program being the mutual contrast for comparison are presented in a forest plot (Figure 5). SBR, pw, and gaitR demonstrated the largest mean difference vs. NE; however SBR only demonstrated a statistically significant difference when compared to the no-exercise program (mean difference = 2.7, 95% CrI = 1.0 to 4.3). The trace plot, density plot, and Brooks-Gelman-Rubin diagnostic statistics showed good convergence, which signifies our data has converged to a reasonable distribution. Relatively reliable evidence was derived from the statistical consistency between direct and indirect evidence demonstrated by the node-splitting model (*p* > 0.05). According to the sensitivity analysis using a Frequentist framework of NMA, the ranking based on the P-scores showed identical results (Table 4). The results suggest that our main findings regarding the relative effectiveness of each exercise intervention are robust for future decisions.

### Subgroup Analyses

**Figure 6:**
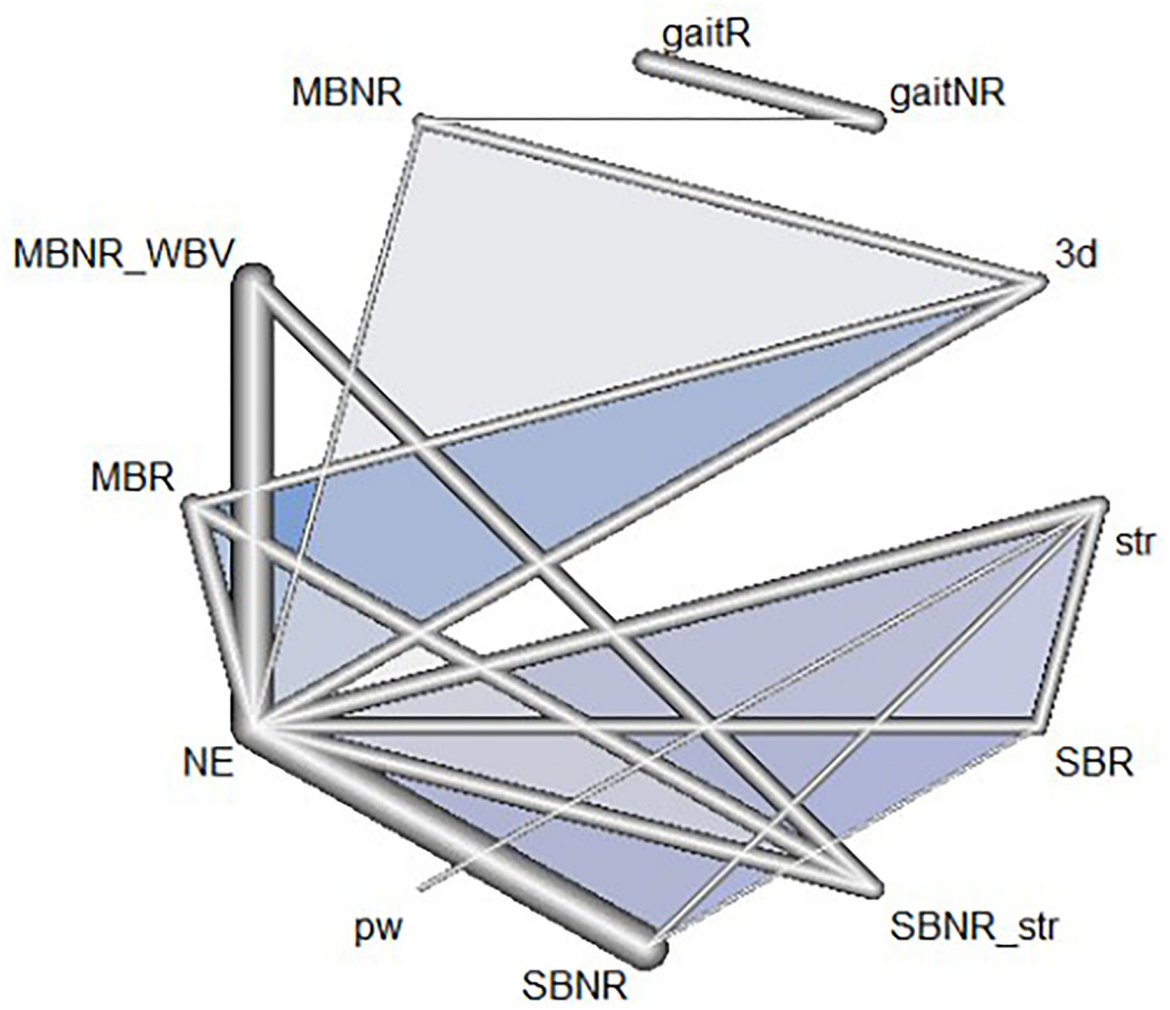
Network geometry of the included exercise programs in healthy older adults: Each line indicates a direct comparison of two different exercise programs. The thickness of the edge is proportional to the number of direct comparisons in the network. Different exercise types combined in one program are connected via underscores. The blue triangles refer to multi-arm trials comprised of three exercise programs in the nodes. SBR, Single balance exercise including reactive balance component; SBNR, Single balance exercise not including reactive balance component; MBR, Multiple balance exercises including reactive balance component; MBNR, Multiple balance exercises not including reactive balance component; gaitR, Gait training including reactive balance component; gaitNR, Gait training not including reactive balance component; WBV, Whole body vibration; str, Strength; pw, Power; 3d, 3D exercise; NE, No exercise.

In the subgroup analysis for healthy older adults (*k* = 29, *n* = 1120, age = 71.5 ± 3.7 years), effects of 12 exercise programs were compared (Figure 6).

**Figure 7:**
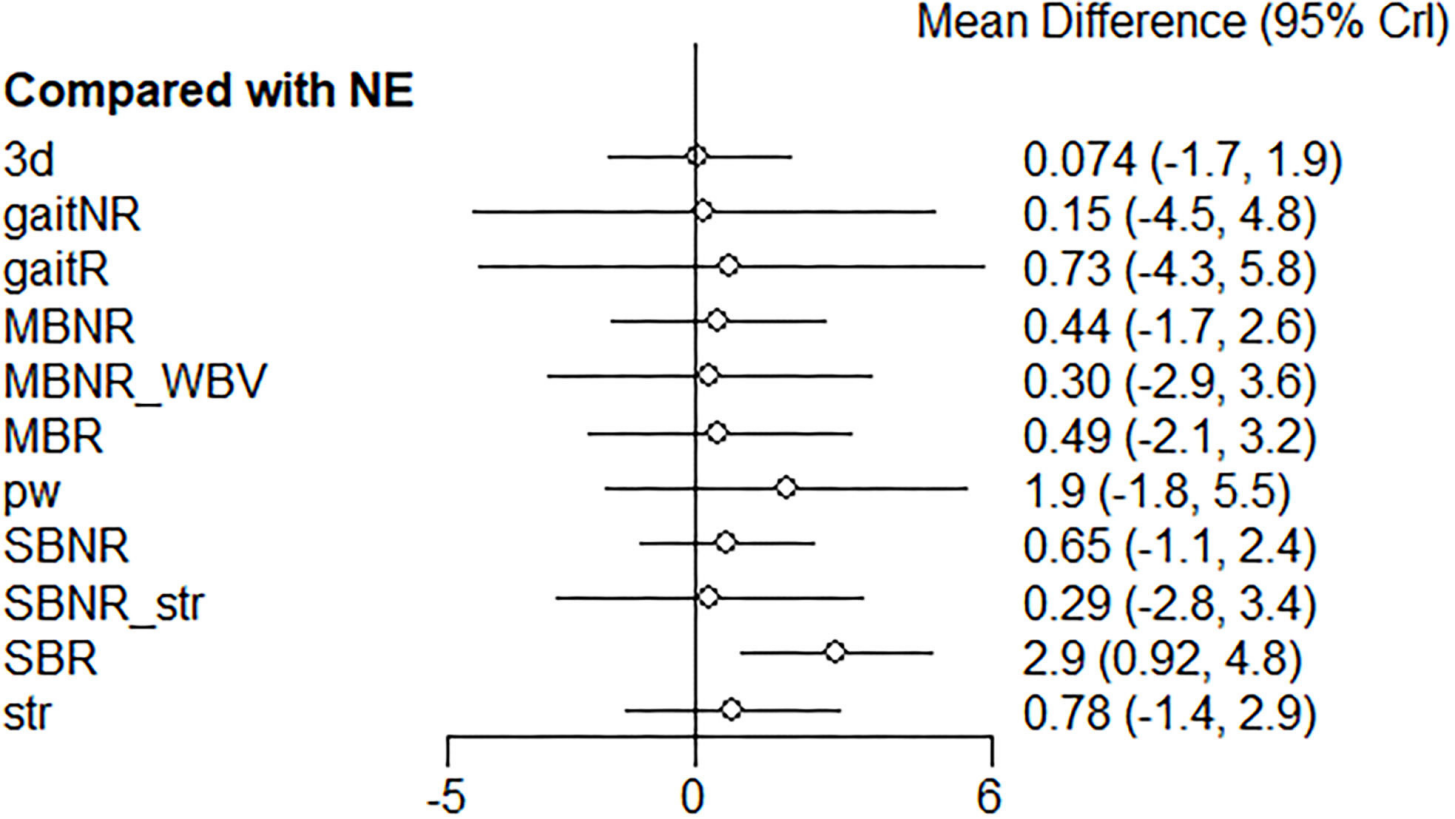
Forest plot of the relative effects of exercise interventions with a no-exercise as a reference group in healthy older adults. SBR, Single balance exercise including reactive balance component; SBNR, Single balance exercise not including reactive balance component; MBR, Multiple balance exercises including reactive balance component; MBNR, Multiple balance exercises not including reactive balance component; gaitR, Gait training including reactive balance component; gaitNR, Gait training not including reactive balance component; WBV, Whole body vibration; str, Strength; pw, Power; 3d, 3D exercise; NE, No exercise.

**Figure 8:**
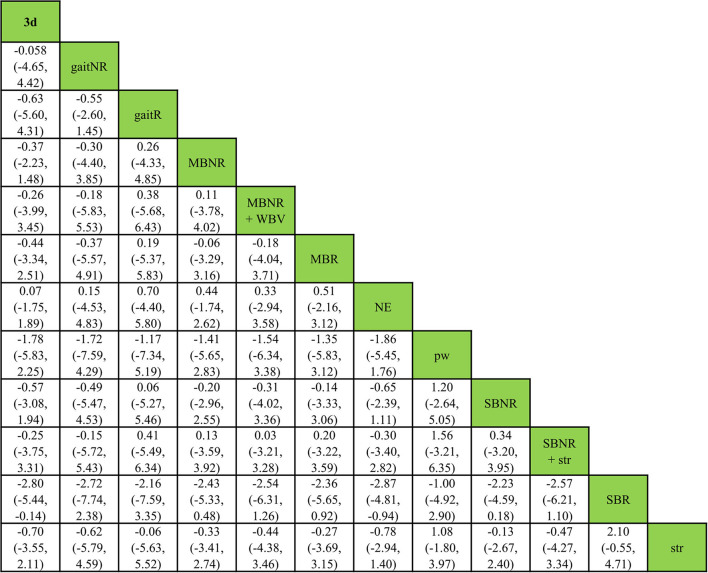
Relative effect estimates with 95% credible intervals of all pairs of exercise interventions in healthy older adults.

According to the SUCRA scores, SBR was the highest-ranked exercise program (0.90), followed by pw (0.71), which was consistent with the ranking in the complete sample (Table 5). The other exercise programs ranked slightly differently from the NMA for the complete sample; however, the rankings based on the SUCRA scores were consistent with those estimated by P-scores in the frequentist framework (Table 5). The relative effects of all exercise interventions compared to NE were presented in Figure 7. A relative effect matrix was additionally created for all comparisons in the healthy older adults (Figure 8). Too few trials in other disease categories (Parkinson's disease: 6 trials, arthritis: 2 trials, osteopenia: 1 trial) and types of exercise interventions were available to establish a network in each category and conduct further disease-specific subgroup analyses.

**Table 5:** Ranking of exercise interventions in healthy older adults.

**Bayesian framework**			**Frequentist framework**		
**Ranking**	**Exercise**	**SUCRA score**	**Ranking**	**Exercise**	**P-score**
1	SBR	0.90	1	SBR	0.95
2	pw	0.71	2	pw	0.76
3	str	0.52	3	str	0.53
4	gaitR	0.52	4	gaitR	0.52
5	SBNR	0.50	5	SBNR	0.52
6	MBR	0.47	6	MBR	0.47
7	MBNR	0.46	7	MBNR	0.46
8	MBNR + WBV	0.43	8	MBNR + WBV	0.41
9	SBNR + str	0.42	9	SBNR + str	0.41
10	gaitNR	0.40	10	gaitNR	0.37
11	3d	0.35	11	3d	0.32
12	NE	0.32	12	NE	0.28

*SBR, Single balance exercise including reactive balance component; SBNR, Single balance exercise not including reactive balance component; MBR, Multiple balance exercises including reactive balance component; MBNR, Multiple balance exercises not including reactive balance component; gaitR, Gait training including reactive balance component; gaitNR, Gait training not including reactive balance component; WBV, Whole body vibration; str, Strength; pw, Power; 3d, 3D exercise; NE, No exercise*.

**Figure 9:**
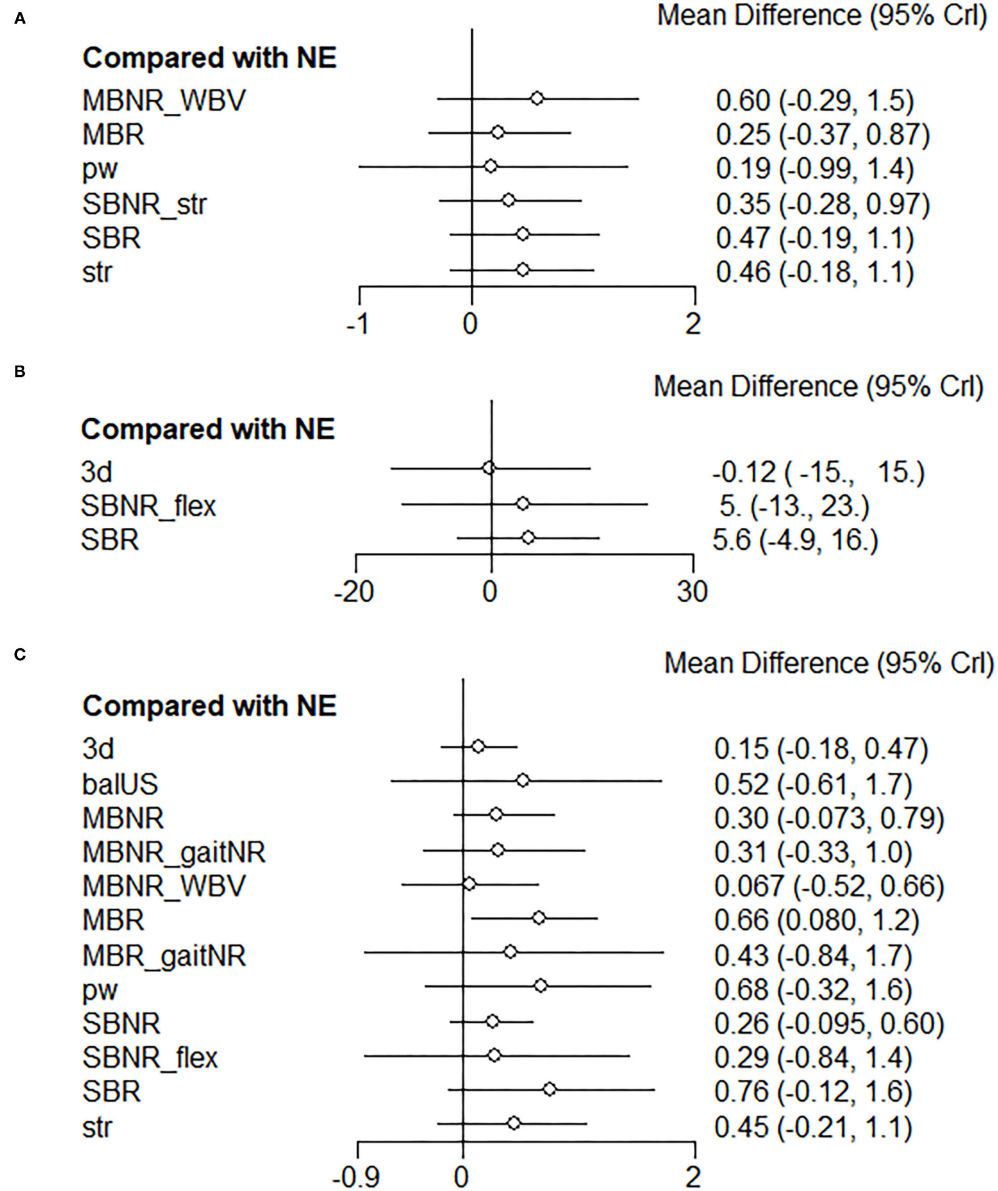
Forest plots of the relative effects of exercise interventions with a no-exercise as a reference group in each reactive balance outcome category. **(A)** Simulated forward falls, **(B)** Being pushed or pulled, and **(C)** Movable platform. SBR, Single balance exercise including reactive balance component; SBNR, Single balance exercise not including reactive balance component; MBR, Multiple balance exercises including reactive balance component; MBNR, Multiple balance exercises not including reactive balance component; WBV, Whole body vibration; str, Strength; pw, Power; 3d, 3D exercise; NE, No exercise.

For the second subgroup analysis regarding the types of reactive balance outcomes, the first type (simulated slip or trip while walking) was analyzed for gaitR vs. gaitNR using a multilevel MA due to insufficient trials in other treatment comparisons (i.e., only one direct comparison). The second (simulated forward falls), third (being pushed or pulled), and fourth (movable platform) types were analyzed using NMA. The fifth type (balance test battery) was not analyzed due to the insufficient number of exercise interventions and direct comparisons to establish a network. When a slip or trip was simulated while walking, participants showed greater improvements in measures of balance recoveries after gaitR training vs. gaitNR training (SMD = 0.60; 95% CI, 0.33 to 0.88). In other types, SBR presented the first or second highest probability of being the best intervention for improving each reactive balance task. The ranking and relative effects of each exercise vs. NE are reported in Table 6 and Figure 9, respectively.

**Table 6:** Ranking of exercise interventions in each reactive balance outcome category.

**A**			**B**			**C**		
**Ranking**	**Exercise**	**SUCRA score**	**Ranking**	**Exercise**	**SUCRA score**	**Ranking**	**Exercise**	**SUCRA score**
1	MBNR + WBV	0.77	1	SBR	0.73	1	SBR	0.79
2	SBR	0.65	2	SBNR + flex	0.63	2	MBR	0.75
3	Str	0.64	3	3d	0.35	3	pw	0.72
4	SBNR + str	0.52	4	NE	0.30	4	balUS	0.60
5	pw	0.39				5	str	0.58
6	MBR	0.39				6	MBR +gaitNR	0.54
7	NE	0.14				7	MBNR	0.48
						8	MBNR +gaitNR	0.48
						9	SBNR	0.43
						10	SBNR + flex	0.43
						11	3d	0.30
						12	MBNR + WBV	0.25
						13	NE	0.14

*A. Simulated forward falls, B. Being pushed or pulled, C. Movable platform. SBR, Single balance exercise including reactive balance component; SBNR, Single balance exercise not including reactive balance component; MBR, Multiple balance exercises including reactive balance component; MBNR, Multiple balance exercises not including reactive balance component; WBV, Whole body vibration; str, Strength; pw, Power; 3d, 3D exercise; NE, No exercise*.

## Discussion

To our knowledge, this study is the first NMA to determine which type of exercise intervention is most effective in improving reactive balance in older adults. In this study, we compared the effects of commonly used exercise interventions on reactive balance in older adults. The NMA was used to analyze the data of 39 RCTs including 1,388 participants, which revealed that older adults receiving a balance exercise with a reactive balance component showed the most improvements in reactive balance, followed by power training (second) and gait training with a reactive balance component (third) among 17 different exercise interventions.

The results of this current study highlight the importance of applying the principle of specificity to training interventions designed to improve reactive balance. This is consistent with the hypothesis put forth by Grabiner et al. (2014) suggesting that task-specific perturbation training is superior to conventional exercise approaches in improving reactive balance capacity and thus preventing falls (Grabiner et al., 2014). Of the 46 trials in the current study, there were 20 trials including at least one exercise intervention with a reactive balance component, and ten of which utilized the same parameters of postural perturbations during the training and assessment (i.e., task-specific reactive balance training) (Wolf et al., 1997; Bieryla et al., 2007; Beling and Roller, 2009; Mansfield et al., 2010; Parijat and Lockhart, 2012; Morat et al., 2019; Okubo et al., 2019; Allin et al., 2020; Arghavani et al., 2020; Rieger et al., 2020). This latter point is especially important given that a specific type of reactive balance exercise has no, or at most a limited transfer effect on non-trained reactive balance tasks (Kümmel et al., 2016; Harper et al., 2021). The cognitive processes, muscle synergies, and succeeding kinematic strategies to counteract the perturbation are entirely determined by the parameters of the perturbations, such as type, magnitude, direction, and the point of application (Winter et al., 1990; Grabiner et al., 2014; Chen et al., 2017), and reactive balance improves in the tasks that are specifically trained with the same parameters. That is one of the reasons SBR showed greater improvements than other types of exercises. It seems reasonable to speculate that if all 20 trials used the exact same training and assessment tasks, the performance gains in reactive balance would be even greater. However, the estimates in the second subgroup analyses (i.e., types of reactive balance outcomes) regarding high SUCRA scores of SBR should be interpreted with caution given the small number of trials and several wide credible intervals.

VRepeated exposure to specific learning environments, therefore, leads to specific motor adaptation and learning. Motor adaptation is a learning process in which the nervous system learns how to predict and cancel impacts of a novel environment (e.g., perturbation), and ultimately maximize performance in that environment (Izawa et al., 2008). The cerebral cortex plays a key role in the acquisition and facilitation of balance recovery skills (Beck et al., 2007; Bolton, 2015). Through repeated exposure to a postural perturbation, our sensorimotor system learns (e.g., procedural learning) internal models for the sensorial prediction and motor commands and uses the learned models for an efficient and optimized movement plan (Izawa et al., 2008), ultimately improving compensatory reactions in older adults (Bohm et al., 2015; König et al., 2019). If mechanical perturbations transpire in consistent patterns with regards to the timing, magnitude, type, and direction, those who have undergone training using the same perturbation system could employ proactive (anticipatory) postural adjustments (i.e., feedforward control in anticipation of or before a postural perturbation) (Bhatt et al., 2006; Aruin et al., 2017; Curuk et al., 2020). Utilization of proactive postural adjustments, facilitated by the repeated exposure to the perturbation, significantly reduces the need for compensatory adjustments after a perturbation (Kanekar and Aruin, 2014); thus, the predictability regarding the perturbation and reactive balance task ultimately imparts greater adaptability and controllability. Such motor training is capable of altering corticospinal excitations and reorganizing motor maps and synaptic changes in the cerebral cortex, which ultimately facilitates the acquisition of a specific balance recovery skill (Beck et al., 2007; Grabiner et al., 2014), and the neuroplastic changes after training offer revealing clinical insights. However, when the patterns of a perturbation unpredictably change, the proactive postural adjustments, that are strictly relying on prior experience, can be deteriorated, which may compromise application to real-world falls where people rarely know in advance how and when they will get perturbed. Thus, a perturbation during training needs to be offered in various patterns to maximize the unpredictability and prepare older adults for the unpredictable nature of real-world falls (Harper et al., 2021). Further, to promote motor adaptation and learning, the elements of the training regimen should be properly determined first, and the challenge should be increased by adjusting the parameters of the perturbation, complexity of the context, and cognitive processing demands (Harper et al., 2021).

The greatest effect of SBR and relatively less effective multicomponent exercise interventions can be further scrutinized via several critical principles of exercise training including volume, intensity, and frequency. Training volume is largely determined by the time commitment (duration) of the training. However, the total duration and frequency of the interventions are broadly ranged across the included studies as previously reported, and the average duration of each training session was 52.2 ± 19.7 min. If an intervention included multiple types of exercises in a single session, the intervention may lack the critical time needed to focus on reactive balance training. According to Burgomaster et al. (Burgomaster et al., 2008), low-volume, high-intensity training and high-volume, low-intensity training induce comparable changes in selected whole-body and skeletal muscle adaptations when the frequencies and the total durations are identical (Burgomaster et al., 2008; Hawley, 2008). Thus, if lack of time is a barrier to satisfying the need for reactive balance training, the intensity aspect of the training should be considered as a way to compensate for the deficit and induce targeted changes in reactive balance. Further, it is encouraging that Bhatt and Pai have demonstrated significant improvements in reactive balance performance after a single high-intensity training with task-specific postural perturbations (Bhatt and Pai, 2009). This is particularly noteworthy given that such minimal training effects were retained for several months when properly selecting the intensity and specificity of the training despite the relatively small total volume. Thus, future trials may wish to take account of the aforementioned factors, including specificity, volume, and intensity of the training to maximize the time-effective transfer to real-world scenarios.

Lastly, given the high ranking of power training, the probable inter-relation with reactive balance is clinically notable. In situations where a mechanical perturbation is applied and a fall begins, the rate of torque development in the lower or upper extremity joints with intersegment coordination has been considered as a critical determinant of balance recovery by taking a step or reach to grasp (Madigan, 2006). Aging inherently brings a loss of motor neurons, associated with apoptosis, and reduction and denervation of muscle fibers, specifically related to type II muscle fibers. These changes lead to a decrease in the muscles' capacity to produce maximum muscle strength, power, and rate of force development (Aagaard et al., 2010). In general, fallers generate less muscle power than non-fallers, and older adults generate less power than young adults (Madigan, 2006; Perry et al., 2007). By utilizing the comparability between muscle power and reactive balance, such as forceful and controlled movements with high velocity, all power training groups in the current analysis demonstrated improvements in measures of reactive balance. There are a handful of studies investigating the correlations between muscle power and reactive balance performances (Muehlbauer et al., 2015); however, the effectiveness of power training on reactive balance has been explored only in a few, recent trials (Pamukoff et al., 2014; Inacio et al., 2018; Cherup et al., 2019). The results of this study may have implications for future directions in assessing the relationship between muscle power and reactive balance.

Given that the vast majority of falls occur while walking (Tinetti et al., 1988; Berg et al., 1997; Li et al., 2006; Kelsey et al., 2012; Robinovitch et al., 2013), training to counter postural perturbations while walking is imperative. However, the ranking of gaitR was relatively lower than SBR. Because gaitR was only compared with gaitNR in the network, the ranking was dominantly determined by indirect evidence. Standard errors from indirect evidence are greater than those from direct evidence, which represents the lower accuracy of an estimate (Higgins et al., 2019). Thus, the indirect evidence should be interpreted with caution, and more RCTs with direct comparisons between gaitR and other exercises may guarantee more accurate posterior distributions and the ranking of gaitR.

### Clinical Implications

Considering the findings of this study, it would be advisable for clinicians to preferentially include reactive balance training in line with specifically targeted context, direction, and type of postural perturbations, and power training as a secondarily or complementary approach to improve reactive balance in older adults irrespective of their clinical classifications. Multicomponent exercise interventions not including a reactive balance component may not bring as marked changes in reactive balance as a single reactive balance training does, whereas they still have benefits regarding general health and physical functioning. The possibility of task-specific training adaptations with balance training using external mechanical perturbations has far-reaching clinical and research implications. In fact, beyond simply training one specific type of balance reaction (e.g., a slip), future trials may wish to include multiple types of reactive balance tasks in various simulated contexts that are likely to occur in daily life and appraise the generalizability and ecological validity of the trained tasks from a long-term perspective. Moreover, the addition of power training may synergize the effects on functional reflex activities as well as general functional capabilities needed for daily tasks and reducing falls in older adults.

### Strengths and Limitations

One of the major strengths of the current study is the use of a NMA. The notable advantage of a NMA over a conventional pairwise meta-analysis is the ability to allow for indirect comparisons, accounting for the effects of multiple interventions in a single statistical model (Schwarzer et al., 2015). Thus, a NMA concurrently summarizes both direct and indirect comparisons between multifarious interventions and enables more complex statistical models and broader interpretation. Random-effects models attempt to generalize the results beyond the trials included in the NMA with an assumption that the selected trials are random samples from a larger population (Cheung et al., 2012). Accordingly, the use of a NMA with a random-effects model in this study enhances the applicability and generalizability of study findings. It should be noted that in general, the indirect estimates tend to have greater variance than direct estimates, and the reliability of the indirect estimates are influenced by the number of direct estimates in the network (Dias et al., 2018). Future meta-analyses may wish to assess publication bias and heterogeneity with a greater number of trials in each direct comparison.

The interpretations of the results in the current study are limited due to small sample sizes and the existence of the probable risk of bias in the included studies. For example, only two trials included more than 100 total participants (Bogaerts et al., 2007; Wang et al., 2019). Furthermore, there was heterogeneity in participants and exercise interventions. For example, there were several distinct disease groups, and the frequency and duration were set differently for various exercise interventions pooled together. With further trials, future reviews may wish to break down the analyses on the basis of hypothetical effect modifiers, such as detailed age and disease groups, baseline functional capacities, or dosage of intervention, for more specific clinical decisions. Also, the low number of trials per comparison precluded investigating sources of publication bias and heterogeneity, and the overall risk of bias was appraised as some concern or high-risk level. Thus, a comprehensive search of published and unpublished works of literature with a paired screening process was conducted to guarantee all available literature was identified to reduce the potential risk of publication bias. Considering the number of trials per each direct comparison, sample sizes, and overall risks of bias, thei results of our analyses may as such guide future research. Despite the aforementioned limitations, we believe that this systematic review with a NMA shed light on better understanding effective interventions for reactive balance in older adults via more comprehensive and inclusive analyses of available literature.

## Conclusions

In conclusion, our NMA indicates that SBR, which simulates a real-life fall scenario and induces a specific balance recovery, is generally more efficacious in improving reactive balance than any other exercise intervention in older adults. Importantly, power training also appears to have greater impacts on reactive balance than other exercise interventions. Our results highlight the importance of task-specific exercise interventions with respect to the targeted postural perturbation and reactions. More trials with high methodological quality, low risk of bias, larger samples, and older adults with a specific disease or disability need to be conducted to construct a comprehensive literature basis, which would facilitate a more thorough NMA. The findings of this study could be used to design exercise-based interventions for improving reactive balance in older adults.

## Data Availability Statement

The original contributions presented in the study are included in the article/Supplementary Material, further inquiries can be directed to the corresponding author/s.

## Author Contributions

YK, MV, and EB performed the search, selection, data extraction, and risk of bias assessments. YK performed all data analyses, drafted the manuscript, and attests that all listed authors meet the authorship criteria and that no others meeting the criteria have been omitted. All authors participated in the conception and design of the study, provided feedback on early and advanced drafts of the manuscript, critically revised for important intellectual content, approved the final version of the manuscript, and agreed to be accountable for all aspects of the work.

## Conflict of Interest

The authors declare that the research was conducted in the absence of any commercial or financial relationships that could be construed as a potential conflict of interest.

## Publisher's Note

All claims expressed in this article are solely those of the authors and do not necessarily represent those of their affiliated organizations, or those of the publisher, the editors and the reviewers. Any product that may be evaluated in this article, or claim that may be made by its manufacturer, is not guaranteed or endorsed by the publisher.

## References

[B1] Aagaard P. Suetta C. Caserotti P. Magnusson S. P. Kjær M. (2010). Role of the nervous system in sarcopenia and muscle atrophy with aging: strength training as a countermeasure. Scandinav. J. Med. Sci. Sport. 20, 49–64. 10.1111/j.1600-0838.2009.01084.x 20487503

[B2] Alissa N. Akinlosotu R. Y. Shipper A. G. Wheeler L. A. Westlake K. P. (2020). A systematic review of upper extremity responses during reactive balance perturbations in aging. Gait. Posture. 82, 138–146. 10.1016/j.gaitpost.2020.08.134 32927220

[B3] Allin L. J. Brolinson P. G. Beach B. M. Kim S. Nussbaum M. A. Roberto K. A. . (2020). Perturbation-based balance training targeting both slip- and trip-induced falls among older adults: a randomized controlled trial. BMC Geriatr. 20, 205. 10.1186/s12877-020-01605-9 32532221 PMC7291462

[B4] Ambrose A. F. Paul G. Hausdorff J. M. (2013). Risk factors for falls among older adults: A review of the literature. Maturitas. 75, 51–61. 10.1016/j.maturitas.2013.02.009 23523272

[B5] Arampatzis A. Peper A. Bierbaum S. (2011). Exercise of mechanisms for dynamic stability control increases stability performance in the elderly. J. Biomech. 44, 52–58. 10.1016/j.jbiomech.2010.08.023 20832803

[B6] Arghavani H. Zolaktaf V. Lenjannejadian S. (2020). Comparing the effects of anticipatory postural adjustments focused training and balance training on postural preparation, balance confidence and quality of life in elderly with history of a fall. Aging Clin. Exper. Res. 32, 1757–1765. 10.1007/s40520-019-01358-5 31608424

[B7] Aruin A. S. Ganesan M. Lee Y. (2017). Improvement of postural control in individuals with multiple sclerosis after a single-session of ball throwing exercise. Mult. Scleros. Relat. Disord. 17, 224–229. 10.1016/j.msard.2017.08.013 29055463

[B8] Beck S. Taube W. Gruber M. Amtage F. Gollhofer A. Schubert M. (2007). Task-specific changes in motor evoked potentials of lower limb muscles after different training interventions. Brain Res. 1179, 51–60. 10.1016/j.brainres.2007.08.048 17889840

[B9] Beling J. Roller M. (2009). Multifactorial intervention with balance training as a core component among fall-prone older adults. J. Geriatr. Phys. Ther. 32, 125–133. 10.1519/00139143-200932030-00008 20128337

[B10] Berg W. P. Alessio H. M. Mills E. M. Tong C. (1997). Circumstances and consequences of falls in independent community-dwelling older adults. Age Ageing. 26, 261–268. 10.1093/ageing/26.4.261 9271288

[B11] Bhatt T. Pai Y.-C. (2009). Prevention of slip-related backward balance loss: the effect of session intensity and frequency on long-term retention. Arch. Phys. Med. Rehabil. 90, 34–42. 10.1016/j.apmr.2008.06.021 19154827 PMC2677700

[B12] Bhatt T. Wening J. D. Pai Y.-C. (2006). Adaptive control of gait stability in reducing slip-related backward loss of balance. Exp. Brain Res. 170, 61–73. 10.1007/s00221-005-0189-5 16344930

[B13] Bieryla K. A. Madigan M. L. Nussbaum M. A. (2007). Practicing recovery from a simulated trip improves recovery kinematics after an actual trip. Gait. Posture. 26, 208–213. 10.1016/j.gaitpost.2006.09.010 17046260

[B14] Bogaerts A. Verschueren S. Delecluse C. Claessens A. L. Boonen S. (2007). Effects of whole body vibration training on postural control in older individuals: a 1 year randomized controlled trial. Gait. Posture. 26, 309–316. 10.1016/j.gaitpost.2006.09.078 17074485

[B15] Bohm S. Mademli L. Mersmann F. Arampatzis A. (2015). Predictive and reactive locomotor adaptability in healthy elderly: A systematic review and meta-analysis. Sports Med. 45, 1759–1777. 10.1007/s40279-015-0413-9 26487633 PMC4656697

[B16] Bolton D. A. E. (2015). The role of the cerebral cortex in postural responses to externally induced perturbations. Neurosci. Biobehav Rev. 57, 142–155. 10.1016/j.neubiorev.2015.08.014 26321589

[B17] Borenstein M. Hedges L. V. Higgins J. P. T. Rothstein H. R. (2009). Introduction to Meta-Analysis. John Wiley and Sons. 10.1002/978047074338629218357

[B18] Brooks S. P. Gelman A. (1998). General methods for monitoring convergence of iterative simulations. Null. 7, 434–455. 10.1080/10618600.1998.10474787

[B19] Bruderer-Hofstetter M. Rausch-Osthoff A.-K. Meichtry A. Münzer T. Niedermann K. (2018). Effective multicomponent interventions in comparison to active control and no interventions on physical capacity, cognitive function and instrumental activities of daily living in elderly people with and without mild impaired cognition – A systematic review and network meta-analysis. Age. Res. Rev. 45, 1–14. 10.1016/j.arr.2018.04.002 29679658

[B20] Burgomaster K. A. Howarth K. R. Phillips S. M. Rakobowchuk M. MacDonald M. J. McGee S. L. . (2008). Similar metabolic adaptations during exercise after low volume sprint interval and traditional endurance training in humans. J. Physiol. 586, 151–160. 10.1113/jphysiol.2007.142109 17991697 PMC2375551

[B21] Cabrera-Martos I. Jiménez-Martín A. T. López-López L. Rodríguez-Torres J. Ortiz-Rubio A. Valenza M. C. (2020). Effects of a core stabilization training program on balance ability in persons with Parkinson's disease: a randomized controlled trial. Clin. Rehabil. 34, 764–772. 10.1177/0269215520918631 32349543

[B22] Chen B. Lee Y.-J. Aruin A. S. (2017). Role of point of application of perturbation in control of vertical posture. Exp. Brain Res. 235, 3449–3457. 10.1007/s00221-017-5069-2 28840283

[B23] Cherup N. P. Buskard A. N. L. Strand K. L. Roberson K. B. Michiels E. R. Kuhn J. E. . (2019). Power vs strength training to improve muscular strength, power, balance and functional movement in individuals diagnosed with Parkinson's disease. Exp. Gerontol. 128, 110740. 10.1016/j.exger.2019.110740 31648006

[B24] Cheung M. W.-L. Ho R. C. M. Lim Y. Mak A. (2012). Conducting a meta-analysis: basics and good practices. Int. J. Rheumat. Dis. 15, 129–135. 10.1111/j.1756-185X.2012.01712.x 22462415

[B25] Chyu M.-C. James C. R. Sawyer S. F. Brismée J.-M. Xu K. T. Poklikuha G. . (2010). Effects of tai chi exercise on posturography, gait, physical function and quality of life in postmenopausal women with osteopaenia: A randomized clinical study. Clin. Rehabil. 24, 1080–1090. 10.1177/0269215510375902 20702512

[B26] Curuk E. Lee Y. Aruin A. S. (2020). Individuals with stroke improve anticipatory postural adjustments after a single session of targeted exercises. Human Movem. Sci. 69, 102559. 10.1016/j.humov.2019.102559 31989951

[B27] Deandrea S. Lucenteforte E. Bravi F. Foschi R. La Vecchia C. Negri E. (2010). Risk factors for falls in community-dwelling older people: a systematic review and meta-analysis. Epidemiology. 21, 658–668. 10.1097/EDE.0b013e3181e89905 20585256

[B28] Dias S. Ades A. E. Welton N. J. Jansen J. P. Sutton A. J. (2018). Network Meta-Analysis for Decision-Making. John Wiley and Sons. 10.1002/978111895165125855820

[B29] Donath L. Roth R. Hürlimann C. Zahner L. Faude O. (2016). Pilates vs. balance training in healthy community-dwelling seniors: A 3-arm, randomized controlled trial. Int. J. Sports Med. 37, 202–210. 10.1055/s-0035-1559695 26783852

[B30] Fu R. Vandermeer B. W. Shamliyan T. A. O'Neil M. E. Yazdi F. Fox S. H. . (2008). Handling continuous outcomes in quantitative synthesis. In Methods Guide for Effectiveness and Comparative Effectiveness Reviews AHRQ Methods for Effective Health Care. (Rockville (MD): Agency for Healthcare Research and Quality (US)). Available online at: http://www.ncbi.nlm.nih.gov/books/NBK154408/. (Accessed May 25, 2021). 24006546

[B31] Gatts S. (2008). Neural mechanisms underlying balance control in Tai Chi. Med. Sport Sci. 52, 87–103. 10.1159/000134289 18487889

[B32] Gatts S. K. Woollacott M. H. (2007). How Tai Chi improves balance: biomechanics of recovery to a walking slip in impaired seniors. Gait. Posture. 25, 205–214. 10.1016/j.gaitpost.2006.03.011 16672187

[B33] Grabiner M. D. Crenshaw J. R. Hurt C. P. Rosenblatt N. J. Troy K. L. (2014). Exercise-Based Fall Prevention: Can You Be a Bit More Specific? Exer. Sport Sci. Rev. 42, 161–168. 10.1249/JES.0000000000000023 25062002

[B34] Granacher U. Gollhofer A. Strass D. (2006). Training induced adaptations in characteristics of postural reflexes in elderly men. Gait. Posture. 24, 459–466. 10.1016/j.gaitpost.2005.12.007 16472525

[B35] Granacher U. Gruber M. Gollhofer A. (2009). Resistance training and neuromuscular performance in seniors. Int. J. Sports Med. 30, 652–657. 10.1055/s-0029-1224178 19569007

[B36] Hamed A. Bohm S. Mersmann F. Arampatzis A. (2018). Exercises of dynamic stability under unstable conditions increase muscle strength and balance ability in the elderly. Scand. J. Med. Sci. Sports 28, 961–971. 10.1111/sms.13019 29154407

[B37] Harper S. A. Beethe A. Z. Dakin C. J. Bolton D. A. E. (2021). Promoting Generalized Learning in Balance Recovery Interventions. Brain Sci. 11, 402. 10.3390/brainsci11030402 33810159 PMC8004641

[B38] Hatzitaki V. Voudouris D. Nikodelis T. Amiridis I. G. (2009). Visual feedback training improves postural adjustments associated with moving obstacle avoidance in elderly women. Gait. Posture. 29, 296–299. 10.1016/j.gaitpost.2008.09.011 18996012

[B39] Hawley J. A. (2008). Specificity of training adaptation: time for a rethink? J. Physiol. 586, 1–2. 10.1113/jphysiol.2007.147397 18167367 PMC2375570

[B40] Hespanhol L. Vallio C. S. Costa L. M. Saragiotto B. T. (2019). Understanding and interpreting confidence and credible intervals around effect estimates. Braz. J. Phys. Ther. 23, 290–301. 10.1016/j.bjpt.2018.12.006 30638956 PMC6630113

[B41] Higgins J. P. T. Thomas J. Chandler J. Cumpston M. Li T. Page M. J. . (2019). Cochrane Handbook for Systematic Reviews of Interventions. John Wiley and Sons. 10.1002/978111953660431643080

[B42] Howe T. E. Rochester L. Neil F. Skelton D. A. Ballinger C. (2011). Exercise for improving balance in older people. Cochrane Datab. System. Rev. 10.1002/14651858.CD004963.pub3 22071817 PMC11493176

[B43] Hu M. H. Woollacott M. H. (1994). Multisensory training of standing balance in older adults: II. Kinematic and electromyographic postural responses. J. Gerontol. 49, M62–71. 10.1093/geronj/49.2.m62 8126354

[B44] Hutton B. Salanti G. Caldwell D. M. Chaimani A. Schmid C. H. Cameron C. . (2015). The PRISMA extension statement for reporting of systematic reviews incorporating network meta-analyses of health care interventions: checklist and explanations. Ann. Intern. Med. 162, 777–784. 10.7326/M14-2385 26030634

[B45] Inacio M. Creath R. Rogers M. W. (2018). Low-dose hip abductor-adductor power training improves neuromechanical weight-transfer control during lateral balance recovery in older adults. Clin. Biomech. 60, 127–133. 10.1016/j.clinbiomech.2018.10.018 30343209 PMC6293473

[B46] Izawa J. Rane T. Donchin O. Shadmehr R. (2008). Motor adaptation as a process of reoptimization. J. Neurosci. 28, 2883–2891. 10.1523/JNEUROSCI.5359-07.2008 18337419 PMC2752329

[B47] Jagdhane S. Kanekar N. S. Aruin A. (2016). The effect of a four-week balance training program on anticipatory postural adjustments in older adults: A pilot feasibility study. Curr. Aging Sci. 9, 295–300. 10.2174/1874609809666160413113443 27071477

[B48] Kanekar N. Aruin A. S. (2014). Aging and balance control in response to external perturbations: role of anticipatory and compensatory postural mechanisms. Age (Dordr) 36, 9621. 10.1007/s11357-014-9621-8 24532389 PMC4082574

[B49] Kelsey J. L. Procter-Gray E. Hannan M. T. Li W. (2012). Heterogeneity of falls among older adults: implications for public health prevention. Am. J. Public Health. 102, 2149–2156. 10.2105/AJPH.2012.300677 22994167 PMC3469772

[B50] Kim S. Lockhart T. (2010). Effects of 8 weeks of balance or weight training for the independently living elderly on the outcomes of induced slips. Int. J. Rehabil. Res. 33, 49–55. 10.1097/MRR.0b013e32832e6b5e 19773670 PMC2929758

[B51] Kim Y. Vakula M. N. Waller B. Bressel E. (2020). A systematic review and meta-analysis comparing the effect of aquatic and land exercise on dynamic balance in older adults. BMC Geriatr. 20, 302. 10.1186/s12877-020-01702-9 32842967 PMC7446104

[B52] Klamroth S. Gaßner H. Winkler J. Eskofier B. Klucken J. Pfeifer K. . (2019). Interindividual balance adaptations in response to perturbation treadmill training in persons with Parkinson disease. J. Neurol. Phys. Ther. 43, 224–232. 10.1097/NPT.0000000000000291 31517749

[B53] König M. Epro G. Seeley J. Potthast W. Karamanidis K. (2019). Retention and generalizability of balance recovery response adaptations from trip perturbations across the adult life span. J. Neurophysiol. 122, 1884–1893. 10.1152/jn.00380.2019 31509470

[B54] Kümmel J. Kramer A. Giboin L.-S. Gruber M. (2016). Specificity of balance training in healthy individuals: a systematic review and meta-analysis. Sports Med. 46, 1261–1271. 10.1007/s40279-016-0515-z 26993132

[B55] Lacroix A. Kressig R. W. Muehlbauer T. Gschwind Y. J. Pfenninger B. Bruegger O. . (2016). Effects of a supervised versus an unsupervised combined balance and strength training program on balance and muscle power in healthy older adults: a randomized controlled trial. GER. 62, 275–288. 10.1159/000442087 26645282

[B56] Lai C.-C. Tu Y.-K. Wang T.-G. Huang Y.-T. Chien K.-L. (2018). Effects of resistance training, endurance training and whole-body vibration on lean body mass, muscle strength and physical performance in older people: a systematic review and network meta-analysis. Age Age. 47, 367–373. 10.1093/ageing/afy009 29471456

[B57] Lesinski M. Hortobágyi T. Muehlbauer T. Gollhofer A. Granacher U. (2015). Effects of balance training on balance performance in healthy older adults: a systematic review and meta-analysis. Sports Med. 45, 1721–1738. 10.1007/s40279-015-0375-y 26325622 PMC4656699

[B58] Li J. X. Xu D. Q. Hong Y. (2009). Changes in muscle strength, endurance, and reaction of the lower extremities with Tai Chi intervention. J. Biomech. 42, 967–971. 10.1016/j.jbiomech.2009.03.001 19356761

[B59] Li W. Keegan T. H. M. Sternfeld B. Sidney S. Quesenberry C. P. Kelsey J. L. (2006). Outdoor falls among middle-aged and older adults: a neglected public health problem. Am. J. Public Health. 96, 1192–1200. 10.2105/AJPH.2005.083055 16735616 PMC1483851

[B60] Lunn D. J. Thomas A. Best N. Spiegelhalter D. (2000). WinBUGS - A Bayesian modelling framework: Concepts, structure, and extensibility. Statist. Comput. 10, 325–337. 10.1023/A:1008929526011

[B61] Ma A. W. W. Wang H.-K. Chen D.-R. Chen Y.-M. Chak Y. T. C. Chan J. W. Y. . (2019). Chinese martial art training failed to improve balance or inhibit falls in older adults. Percept. Mot. Skills 126, 389–409. 10.1177/0031512518824945 30803309

[B62] Madigan M. L. (2006). Age-related differences in muscle power during single-step balance recovery. J. Appl. Biomech. 22, 186–193. 10.1123/jab.22.3.186 17215550

[B63] Mahoney J. R. Cotton K. Verghese J. (2019). Multisensory integration predicts balance and falls in older adults. J. Gerontol. Series A 74, 1429–1435. 10.1093/gerona/gly245 30357320 PMC6696711

[B64] Mansfield A. Peters A. L. Liu B. A. Maki B. E. (2010). Effect of a perturbation-based balance training program on compensatory stepping and grasping reactions in older adults: a randomized controlled trial. Phys. Ther. 90, 476–491. 10.2522/ptj.20090070 20167644

[B65] Marigold D. S. Eng J. J. Dawson A. S. Inglis J. T. Harris J. E. Gylfadóttir S. (2005). Exercise leads to faster postural reflexes, improved balance and mobility, and fewer falls in older persons with chronic stroke. J. Am. Geriatr. Soc. 53, 416–423. 10.1111/j.1532-5415.2005.53158.x 15743283 PMC3226796

[B66] McCrum C. Gerards M. H. G. Karamanidis K. Zijlstra W. Meijer K. (2017). A systematic review of gait perturbation paradigms for improving reactive stepping responses and falls risk among healthy older adults. Eur. Rev. Ag. Phys. Activity. 14, 3. 10.1186/s11556-017-0173-7 28270866 PMC5335723

[B67] Moore B. M. Adams J. T. Willcox S. Nicholson J. (2019). The effect of active physical training interventions on reactive postural responses in older adults: a systematic review. J. Aging Phys. Activity. 27, 252–264. 10.1123/japa.2017-0347 29989462

[B68] Morat M. Bakker J. Hammes V. Morat T. Giannouli E. Zijlstra W. . (2019). Effects of stepping exergames under stable versus unstable conditions on balance and strength in healthy community-dwelling older adults: A three-armed randomized controlled trial. Exper. Gerontol. 127, 110719. 10.1016/j.exger.2019.110719 31513877

[B69] Muehlbauer T. Gollhofer A. Granacher U. (2015). Associations between measures of balance and lower-extremity muscle strength/power in healthy individuals across the lifespan: a systematic review and meta-analysis. Sports Med. 45, 1671–1692. 10.1007/s40279-015-0390-z 26412212 PMC4656701

[B70] Ni M. Mooney K. Richards L. Balachandran A. Sun M. Harriell K. . (2014). Comparative impacts of Tai Chi, balance training, and a specially-designed yoga program on balance in older fallers. Arch. Phys. Med. Rehabil. 95, 1620–1628.e30. 10.1016/j.apmr.2014.04.022 24835753

[B71] Ochi A. Abe T. Yamada K. Ibuki S. Tateuchi H. Ichihashi N. (2015). Effect of balance exercise in combination with whole-body vibration on muscle activity of the stepping limb during a forward fall in older women: a randomized controlled pilot study. Arch. Gerontol. Geriatr. 60, 244–251. 10.1016/j.archger.2014.11.011 25482957

[B72] Okubo Y. Schoene D. Caetano M. J. Pliner E. M. Osuka Y. Toson B. . (2021). Stepping impairment and falls in older adults: a systematic review and meta-analysis of volitional and reactive step tests. Ageing Res. Rev. 66, 101238. 10.1016/j.arr.2020.101238 33352293

[B73] Okubo Y. Sturnieks D. L. Brodie M. A. Duran L. Lord S. R. (2019). Effect of reactive balance training involving repeated slips and trips on balance recovery among older adults: a blinded randomized controlled trial. J. Gerontol. 74, 1489–1496. 10.1093/gerona/glz021 30721985

[B74] Osoba M. Y. Rao A. K. Agrawal S. K. Lalwani A. K. (2019). Balance and gait in the elderly: a contemporary review. Laryngosc. Invest. Otolaryngol. 4, 143–153. 10.1002/lio2.252 30828632 PMC6383322

[B75] Pamukoff D. N. Haakonssen E. C. Zaccaria J. A. Madigan M. L. Miller M. E. Marsh A. P. (2014). The effects of strength and power training on single-step balance recovery in older adults: a preliminary study. Clin. Interv. Aging. 9, 697–704. 10.2147/CIA.S59310 24790422 PMC4000185

[B76] Parijat P. Lockhart T. E. (2012). Effects of moveable platform training in preventing slip-induced falls in older adults. Ann. Biomed. Eng. 40, 1111–1121. 10.1007/s10439-011-0477-0 22134467 PMC3319506

[B77] Parijat P. Lockhart T. E. Liu J. (2015a). Effects of perturbation-based slip training using a virtual reality environment on slip-induced falls. Ann. Biomed. Eng. 43, 958–967. 10.1007/s10439-014-1128-z 25245221 PMC4384510

[B78] Parijat P. Lockhart T. E. Liu J. (2015b). EMG and kinematic responses to unexpected slips after slip training in virtual reality. IEEE Transac. On Bio-Med. Eng. 62, 593–599. 10.1109/TBME.2014.2361324 25296401 PMC4390025

[B79] Perry M. C. Carville S. F. Smith I. C. H. Rutherford O. M. Newham D. J. (2007). Strength, power output and symmetry of leg muscles: effect of age and history of falling. Eur. J. Appl. Physiol. 100, 553–561. 10.1007/s00421-006-0247-0 16847676

[B80] Pluchino A. Lee S. Y. Asfour S. Roos B. A. Signorile J. F. (2012). Pilot study comparing changes in postural control after training using a video game balance board program and 2 standard activity-based balance intervention programs. Arch. Phys. Med. Rehabil. 93, 1138–1146. 10.1016/j.apmr.2012.01.023 22414490

[B81] Qutubuddin A. A. Cifu D. X. Armistead-Jehle P. Carne W. McGuirk T. E. Baron M. S. (2007). A comparison of computerized dynamic posturography therapy to standard balance physical therapy in individuals with Parkinson's disease: a pilot study. NeuroRehabilitation 22, 261–265. 17971615

[B82] Rieger M. M. Papegaaij S. Pijnappels M. Steenbrink F. van Dieën J. H. (2020). Transfer and retention effects of gait training with anterior-posterior perturbations to postural responses after medio-lateral gait perturbations in older adults. Clin. Biomech. 75, N.PAG-N.PAG. 10.1016/j.clinbiomech.2020.104988 32174482

[B83] Robinovitch S. N. Feldman F. Yang Y. Schonnop R. Leung P. M. Sarraf T. . (2013). Video capture of the circumstances of falls in elderly people residing in long-term care: an observational study. Lancet. 381, 47–54. 10.1016/S0140-6736(12)61263-X 23083889 PMC3540102

[B84] Rossi L. P. Brandalize M. Pereira R. Silveira Gomes A. R. (2014). The effects of a perturbation-based balance training on neuromuscular recruitment and functional mobility in community-dwelling older women. Top. Geriatr. Rehabil. 30, 256–263. 10.1097/TGR.0000000000000035

[B85] Rücker G. Cates C. J. Schwarzer G. (2017). Methods for including information from multi-arm trials in pairwise meta-analysis. Res. Synth. Method. 8, 392–403. 10.1002/jrsm.1259 28759708

[B86] Salanti G. Giovane C. D. Chaimani A. Caldwell D. M. Higgins J. P. T. (2014). Evaluating the quality of evidence from a network meta-analysis. PLoS ONE. 9, e99682. 10.1371/journal.pone.0099682 24992266 PMC4084629

[B87] Santos S. M. da Silva R. A. Terra M. B. Almeida I. A. de Melo L. B. Ferraz H. B. (2017). Balance versus resistance training on postural control in patients with Parkinson's disease: a randomized controlled trial. Eur. J. Phys. Rehabil. Med. 53, 173–183. 10.23736/S1973-9087.16.04313-6 27879959

[B88] Schlenstedt C. Paschen S. Kruse A. Raethjen J. Weisser B. Deuschl G. (2015). Resistance versus balance training to improve postural control in Parkinson's disease: A randomized rater blinded controlled study. PLoS ONE 10. 10.1371/journal.pone.0140584 26501562 PMC4621054

[B89] Schwarzer G. Carpenter J. R. Rücker G. (2015). Network meta-analysis. In Meta-Analysis with R Use R!, eds. Schwarzer G. Carpenter J. R. Rücker G. (Cham: Springer International Publishing) p. 187–216. 10.1007/978-3-319-21416-0

[B90] Shimada H. Uchiyama Y. Kakurai S. (2003). Specific effects of balance and gait exercises on physical function among the frail elderly. Clin. Rehabil. 17, 472–479. 10.1191/0269215503cr638oa 12952151

[B91] Shumway-Cook A. Woollacott M. H. (2017). Motor Control: Translating Research Into Clinical Practice. Lippincott Williams and Wilkins.

[B92] Sibley K. M. Thomas S. M. Veroniki A. A. Rodrigues M. Hamid J. S. Lachance C. C. . (2021). Comparative effectiveness of exercise interventions for preventing falls in older adults: a secondary analysis of a systematic review with network meta-analysis. Exper. Gerontol. 143, 111151. 10.1016/j.exger.2020.111151 33186739

[B93] Sohn J. Kim S. (2015). Falls study: Proprioception, postural stability, and slips. Biomed. Mater. Eng. 26, S693–703. 10.3233/BME-151361 26406065

[B94] Sterne J. A. C. Savović J. Page M. J. Elbers R. G. Blencowe N. S. Boutron I. . (2019). RoB 2: a revised tool for assessing risk of bias in randomised trials. BMJ. 366, l4898. 10.1136/bmj.l4898 31462531

[B95] Sutton A. J. Abrams K. R. Jones D. R. Sheldon T. A. Song F. (2000). Methods for Meta-Analysis in Medical Research. John Wiley and Sons.

[B96] Thomas M. Kalicinski M. (2016). The Effects of Slackline Balance Training on Postural Control in Older Adults. J. Aging Phys. Act. 24, 393–398. 10.1123/japa.2015-0099 26583953

[B97] Tinetti M. E. Speechley M. Ginter S. F. (1988). Risk Factors for Falls among Elderly Persons Living in the Community. New Engl. J. Med. 319, 1701–1707. 10.1056/NEJM198812293192604 3205267

[B98] Wang Y. Bhatt T. Liu X. Wang S. Lee A. Wang E. . (2019). Can treadmill-slip perturbation training reduce immediate risk of over-ground-slip induced fall among community-dwelling older adults? J. Biomech. 84, 58–66. 10.1016/j.jbiomech.2018.12.017 30616984 PMC6361674

[B99] Winter D. A. Patla A. E. Frank J. S. (1990). Assessment of balance control in humans. Med. Prog. Technol. 16, 31–51. 2138696

[B100] Wolf S. L. Barnhart H. X. Ellison G. L. Coogler C. E. Atlanta F. I. C. S. I. T. Group (1997). The effect of Tai Chi Quan and computerized balance training on postural stability in older subjects. Phys. Therapy. 77, 371–381. 10.1093/ptj/77.4.371 9105340

[B101] Wooten S. V. Signorile J. F. Desai S. S. Paine A. K. Mooney K. (2018). Yoga meditation (YoMed) and its effect on proprioception and balance function in elders who have fallen: A randomized control study. Complement Ther. Med. 36, 129–136. 10.1016/j.ctim.2017.12.010 29458919

[B102] World Health Organization (2008). WHO Global Report on Falls Prevention in Older Age. World Health Organization.

[B103] Wu P.-Y. Huang K.-S. Chen K.-M. Chou C.-P. Tu Y.-K. (2021). Exercise, nutrition, and combined exercise and nutrition in older adults with sarcopenia: a systematic review and network meta-analysis. Maturitas. 145, 38–48. 10.1016/j.maturitas.2020.12.009 33541561

