# Supplementary material for: Which Exercise Interventions Can Most Effectively Improve Reactive Balance in Older Adults? A Systematic Review and Network Meta-Analysis
Source: Front Aging Neurosci. 2022 Jan 18;13:764826. doi: 10.3389/fnagi.2021.764826 (PMC8804322; doi:10.3389/fnagi.2021.764826)
Supplement: Supplementary file 1 [file Table_1.pdf]

Supplementary Material

Contents

Supplementary Table 1. List of all included studies (n = 46) 2

Supplementary Table 2. Summary of outcome measures and main findings 7

Supplementary Table 3. Summary table of the reviewers' judgements for the risk of bias of each study.. 14

Supplementary Table 1. List of all included studies (n = 46)

Allin, L. J., Brolinson, P. G., Beach, B. M., Kim, S., Nussbaum, M. A., Roberto, K. A., et al. (2020). Perturbation-based balance training targeting both slip- and trip-induced falls among older adults: a randomized controlled trial. *BMC geriatrics* 20, 205. doi:10.1186/s12877-020-01605-9.

Arampatzis, A., Peper, A., and Bierbaum, S. (2011). Exercise of mechanisms for dynamic stability control increases stability performance in the elderly. *J Biomech* 44, 52–58. doi:10.1016/j.jbiomech.2010.08.023.

Arghavani, H., Zolaktaf, V., and Lenjannejadian, S. (2020). Comparing the effects of anticipatory postural adjustments focused training and balance training on postural preparation, balance confidence and quality of life in elderly with history of a fall. *Aging clinical and experimental research* 32, 1757–1765. doi:10.1007/s40520-019-01358-5.

Beling, J., and Roller, M. (2009). Multifactorial intervention with balance training as a core component among fall-prone older adults. *J Geriatr Phys Ther* 32, 125–133. doi:10.1519/00139143-200932030-00008.

Bieryla, K. A., Madigan, M. L., and Nussbaum, M. A. (2007). Practicing recovery from a simulated trip improves recovery kinematics after an actual trip. *Gait Posture* 26, 208–213. doi:10.1016/j.gaitpost.2006.09.010.

Bogaerts, A., Verschueren, S., Delecluse, C., Claessens, A. L., and Boonen, S. (2007). Effects of whole body vibration training on postural control in older individuals: a 1 year randomized controlled trial. *Gait Posture* 26, 309–316. doi:10.1016/j.gaitpost.2006.09.078.

Cabrera-Martos, I., Jiménez-Martín, A. T., López-López, L., Rodríguez-Torres, J., Ortiz-Rubio, A., and Valenza, M. C. (2020). Effects of a core stabilization training program on balance ability in persons with Parkinson's disease: a randomized controlled trial. *Clinical rehabilitation* 34, 764–772. doi:10.1177/0269215520918631.

Cherup, N. P., Buskard, A. N. L., Strand, K. L., Roberson, K. B., Michiels, E. R., Kuhn, J. E., et al. (2019). Power vs strength training to improve muscular strength, power, balance and functional movement in individuals diagnosed with Parkinson's disease. *Exp Gerontol* 128, 110740. doi:10.1016/j.exger.2019.110740.

Chyu, M.-C., James, C. R., Sawyer, S. F., Brismée, J.-M., Xu, K. T., Poklikuha, G., et al. (2010). Effects of tai chi exercise on posturography, gait, physical function and quality of life in postmenopausal women with osteopaenia: A randomized clinical study. *Clinical Rehabilitation* 24, 1080–1090. doi:10.1177/0269215510375902.

Donath, L., Roth, R., Hürlimann, C., Zahner, L., and Faude, O. (2016). Pilates vs. balance training in healthy community-dwelling seniors: A 3-arm, randomized controlled trial. *International journal of sports medicine* 37, 202–210. doi:10.1055/s-0035-1559695.

Gatts, S. (2008). Neural mechanisms underlying balance control in Tai Chi. *Med Sport Sci* 52, 87–103. doi:10.1159/000134289.

Gatts, S. K., and Woollacott, M. H. (2007). How Tai Chi improves balance: biomechanics of recovery to a walking slip in impaired seniors. *Gait & posture* 25, 205–214. doi:10.1016/j.gaitpost.2006.03.011.

Granacher, U., Gollhofer, A., and Strass, D. (2006). Training induced adaptations in characteristics of postural reflexes in elderly men. *Gait Posture* 24, 459–466. doi:10.1016/j.gaitpost.2005.12.007.

Granacher, U., Gruber, M., and Gollhofer, A. (2009). Resistance training and neuromuscular performance in seniors. *Int J Sports Med* 30, 652–657. doi:10.1055/s-0029-1224178.

Hamed, A., Bohm, S., Mersmann, F., and Arampatzis, A. (2018). Exercises of dynamic stability under unstable conditions increase muscle strength and balance ability in the elderly. *Scandinavian journal of medicine & science in sports* 28, 961–971. doi:10.1111/sms.13019.

Hatzitaki, V., Voudouris, D., Nikodelis, T., and Amiridis, I. G. (2009). Visual feedback training improves postural adjustments associated with moving obstacle avoidance in elderly women. *Gait & posture* 29, 296–299. doi:10.1016/j.gaitpost.2008.09.011.

Hu, M. H., and Woollacott, M. H. (1994). Multisensory training of standing balance in older adults: II. Kinematic and electromyographic postural responses. *J Gerontol* 49, M62-71. doi:10.1093/geronj/49.2.m62.

Inacio, M., Creath, R., and Rogers, M. W. (2018). Low-dose hip abductor-adductor power training improves neuromechanical weight-transfer control during lateral balance recovery in older adults. *Clin Biomech (Bristol, Avon)* 60, 127–133. doi:10.1016/j.clinbiomech.2018.10.018.

Jagdhane, S., Kanekar, N., and S. Aruin, A. (2016). The effect of a four-week balance training program on anticipatory postural adjustments in older adults: A pilot feasibility study. *Current Aging Science* 9, 295–300.

Kim, S., and Lockhart, T. (2010). Effects of 8 weeks of balance or weight training for the independently living elderly on the outcomes of induced slips. *Int J Rehabil Res* 33, 49–55. doi:10.1097/MRR.0b013e32832e6b5e.

Klamroth, S., Gaßner, H., Winkler, J., Eskofier, B., Klucken, J., Pfeifer, K., et al. (2019). Interindividual balance adaptations in response to perturbation treadmill training in persons with Parkinson disease. *Journal of Neurologic Physical Therapy* 43, 224–232. doi:10.1097/NPT.0000000000000291.

Lacroix, A., Kressig, R. W., Muehlbauer, T., Gschwind, Y. J., Pfenninger, B., Bruegger, O., et al. (2016). Effects of a supervised versus an unsupervised combined balance and strength

training program on balance and muscle power in healthy older adults: A randomized controlled trial. *GER* 62, 275–288. doi:10.1159/000442087.

Li, J. X., Xu, D. Q., and Hong, Y. (2009). Changes in muscle strength, endurance, and reaction of the lower extremities with Tai Chi intervention. *Journal of Biomechanics* 42, 967–971. doi:10.1016/j.jbiomech.2009.03.001.

Ma, A. W. W., Wang, H.-K., Chen, D.-R., Chen, Y.-M., Chak, Y. T. C., Chan, J. W. Y., et al. (2019). Chinese martial art training failed to improve balance or inhibit falls in older adults. *Perceptual and Motor Skills* 126, 389–409. doi:10.1177/0031512518824945.

Mansfield, A., Peters, A. L., Liu, B. A., and Maki, B. E. (2010). Effect of a perturbation-based balance training program on compensatory stepping and grasping reactions in older adults: A randomized controlled trial. *Phys Ther* 90, 476–491. doi:10.2522/ptj.20090070.

Marigold, D. S., Eng, J. J., Dawson, A. S., Inglis, J. T., Harris, J. E., and Gylfadóttir, S. (2005). Exercise leads to faster postural reflexes, improved balance and mobility, and fewer falls in older persons with chronic stroke. *J Am Geriatr Soc* 53, 416–423. doi:10.1111/j.1532-5415.2005.53158.x.

Morat, M., Bakker, J., Hammes, V., Morat, T., Giannouli, E., Zijlstra, W., et al. (2019). Effects of stepping exergames under stable versus unstable conditions on balance and strength in healthy community-dwelling older adults: A three-armed randomized controlled trial. *Experimental Gerontology* 127, 110719. doi:10.1016/j.exger.2019.110719.

Ni, M., Mooney, K., Richards, L., Balachandran, A., Sun, M., Harriell, K., et al. (2014). Comparative impacts of Tai Chi, balance training, and a specially-designed yoga program on balance in older fallers. *Arch Phys Med Rehabil* 95, 1620-1628.e30. doi:10.1016/j.apmr.2014.04.022.

Ochi, A., Abe, T., Yamada, K., Ibuki, S., Tateuchi, H., and Ichihashi, N. (2015). Effect of balance exercise in combination with whole-body vibration on muscle activity of the stepping limb during a forward fall in older women: a randomized controlled pilot study. *Arch Gerontol Geriatr* 60, 244–251. doi:10.1016/j.archger.2014.11.011.

Okubo, Y., Sturnieks, D. L., Brodie, M. A., Duran, L., and Lord, S. R. (2019). Effect of reactive balance training involving repeated slips and trips on balance recovery among older adults: A blinded randomized controlled trial. *The journals of gerontology. Series A, Biological sciences and medical sciences* 74, 1489–1496. doi:10.1093/gerona/glz021.

Pamukoff, D. N., Haakonssen, E. C., Zaccaria, J. A., Madigan, M. L., Miller, M. E., and Marsh, A. P. (2014). The effects of strength and power training on single-step balance recovery in older adults: a preliminary study. *Clin Interv Aging* 9, 697–704. doi:10.2147/CIA.S59310.

Parijat, P., and Lockhart, T. E. (2012). Effects of moveable platform training in preventing slip-induced falls in older adults. *Annals of biomedical engineering* 40, 1111–1121. doi:10.1007/s10439-011-0477-0.

Parijat, P., Lockhart, T. E., and Liu, J. (2015a). Effects of perturbation-based slip training using a virtual reality environment on slip-induced falls. *Annals of biomedical engineering* 43, 958–967. doi:10.1007/s10439-014-1128-z.

Parijat, P., Lockhart, T. E., and Liu, J. (2015b). EMG and kinematic responses to unexpected slips after slip training in virtual reality. *IEEE transactions on bio-medical engineering* 62, 593–599. doi:10.1109/TBME.2014.2361324.

Pluchino, A., Lee, S. Y., Asfour, S., Roos, B. A., and Signorile, J. F. (2012). Pilot study comparing changes in postural control after training using a video game balance board program and 2 standard activity-based balance intervention programs. *Arch Phys Med Rehabil* 93, 1138–1146. doi:10.1016/j.apmr.2012.01.023.

Qutubuddin, A. A., Cifu, D. X., Armistead-Jehle, P., Carne, W., McGuirk, T. E., and Baron, M. S. (2007). A comparison of computerized dynamic posturography therapy to standard balance physical therapy in individuals with Parkinson's disease: a pilot study. *NeuroRehabilitation* 22, 261–265.

Rieger, M. M., Papegaaij, S., Pijnappels, M., Steenbrink, F., and van Dieën, J. H. (2020). Transfer and retention effects of gait training with anterior-posterior perturbations to postural responses after medio-lateral gait perturbations in older adults. *Clinical Biomechanics* 75, N.PAG-N.PAG. doi:10.1016/j.clinbiomech.2020.104988.

Rossi, L. P., Brandalize, M., Pereira, R., and Silveira Gomes, A. R. (2014). The effects of a perturbation-based balance training on neuromuscular recruitment and functional mobility in community-dwelling older women. *Topics in Geriatric Rehabilitation* 30, 256–263. doi:10.1097/TGR.0000000000000035.

Santos, S. M., da Silva, R. A., Terra, M. B., Almeida, I. A., de Melo, L. B., and Ferraz, H. B. (2017). Balance versus resistance training on postural control in patients with Parkinson's disease: a randomized controlled trial. *Eur J Phys Rehabil Med* 53, 173–183. doi:10.23736/S1973-9087.16.04313-6.

Schlenstedt, C., Paschen, S., Kruse, A., Raethjen, J., Weisser, B., and Deuschl, G. (2015). Resistance versus balance training to improve postural control in Parkinson's disease: A randomized rater blinded controlled study. *PLoS ONE* 10. doi:10.1371/journal.pone.0140584.

Shimada, H., Uchiyama, Y., and Kakurai, S. (2003). Specific effects of balance and gait exercises on physical function among the frail elderly. *Clin Rehabil* 17, 472–479. doi:10.1191/0269215503cr638oa.

Sohn, J., and Kim, S. (2015). Falls study: Proprioception, postural stability, and slips. *Biomed Mater Eng* 26 Suppl 1, S693-703. doi:10.3233/BME-151361.

Thomas, M., and Kalicinski, M. (2016). The effects of slackline balance training on postural control in older adults. *Journal of Aging and Physical Activity* 24, 393–398. doi:10.1123/japa.2015-0099.

Wang, Y., Bhatt, T., Liu, X., Wang, S., Lee, A., Wang, E., et al. (2019). Can treadmill-slip perturbation training reduce immediate risk of over-ground-slip induced fall among community-dwelling older adults? *Journal of biomechanics* 84, 58–66. doi:10.1016/j.jbiomech.2018.12.017.

Wolf, S. L., Barnhart, H. X., Ellison, G. L., Coogler, C. E., and Atlanta FICSIT Group (1997). The effect of Tai Chi Quan and computerized balance training on postural stability in older subjects. *Physical Therapy* 77, 371–381. doi:10.1093/ptj/77.4.371.

Wooten, S. V., Signorile, J. F., Desai, S. S., Paine, A. K., and Mooney, K. (2018). Yoga meditation (YoMed) and its effect on proprioception and balance function in elders who have fallen: A randomized control study. *Complementary therapies in medicine* 36, 129–136. doi:10.1016/j.ctim.2017.12.010.

Supplementary Table 2. Summary of outcome measures and main findings

Study	Reactive balance outcome measures	Outcome variables	Main findings
Allin 2020	Laboratory-induced slip or trip while walking	Slip: peak slip speed, slip distance, non-slipping toe to COM at TD, minimum hip height, margin of stability at TD, velocity of COM relative to BOS at TD, incidence of falls during testing. Trip: trunk angle at TD, recovery step length, minimum hip height, margin of stability, incidence of falls during testing	Regarding slips, several measures of reactive balance and fall incidence were more improved in group 1 versus group 2. No between-group difference regarding trips,
Arampatzis 2011	Simulated forward falls (lean-and-release)	Anterior boundary of the BOS, position of the XCOM, horizontal component of the projection of the COM to the ground, horizontal velocity of the COM, rate of increase of BOS, reaction time, duration until TD, max hip flexion moment, time to max hip moment, rate of hip moment generation, duration of main stance phase	Two exercise groups improved in a similar extent versus group 3.
Arghavani 2020	Pendulum impact received by both hands in the sagittal plane while standing	Muscle onset latencies of TA, MG, RF, BF, RA, ES	Group 1 showed greater rates of progress in all six muscles versus the other two groups. Group 2 showed greater improvements in RF and BF muscles versus group 3.
Beling 2009	Adaptation Test (toes-up and toes-down surface perturbation while standing)	Classified: Adaptive = no falls and less than 2/5 trials in abnormal range; Maladaptive = no falls and greater than 2/5 trials in abnormal range; Unable to Adapt = any fall during the trials	Group 1, but not group 2, showed improvements in both conditions.
Bieryla 2007	Simulated trip while walking	Maximum trunk angle, time to maximum trunk angle, maximum trunk angular velocity, time to maximum trunk angular velocity, trunk angle at foot contact, trunk angle velocity at foot contact, minimum hip height, COM-to-foot distance at foot contact	Group 1 showed a greater reduction in maximum trunk angle and time to maximum trunk angle and increased minimum hip height versus group 2.
Bogaerts 2007	Motor Control Test (unexpected forward and backward platform	Motor Control Test (latency of reaction, response strength), Adaptation Test (capacity to minimize postural sway after the perturbation)	Motor Control Test: Exercise had no effect on latency for any conditions. Adaptation test: Group 1 showed a significant improvement in

	translation while standing), Adaptation Test		the toes-down condition. No group difference in the toes-up condition.
Cabrera-Martos 2020	Mini-BESTest	Reactive postural balance section	Group 1 showed a greater improvement versus group 2.
Cherup 2019	Dynamic posturography (a platform randomly moving in all three planes)	Comprehensive DMA score, time remained on the platform	No significant between-group differences in all outcomes.
Chyu 2010	Motor Control Test, Adaptation Test	Motor Control Test (latency of reaction, magnitude of the postural righting response), Adaptation Test (capacity to minimize postural sway after the perturbation)	No significant between-group differences in all outcomes.
Donath 2016	Platform perturbation (posterior direction) while kneeling	Total COP path length displacement	Two exercise groups showed improvements (greater in the balance group). No improvement in NE group.
Gatts 2007	Laboratory-induced slip while walking	Number of trips and heel strikes during testing, medial cross-step distance, shoulder and trunk angles, COM (velocity, path distance in AP, ML, and vertical directions), COP (velocity, path distance in AP and ML directions), COM-COP separation angles	Group 1, but not group 2, showed significantly reduced tripping, medial cross-step distance, increased use of swing leg heel strike, and COM AP path. In addition, group 1 showed a trend toward increased COM-COP AP angular separation at right heel strike.
Gatts 2008	Laboratory-induced slip while walking	Muscle onset latencies, duration of muscle activities, and duration of co-contraction of TA and MG	Group 1, but not group 2, showed significantly reduced TA response time and decreased co-contraction of antagonist muscles of the perturbed leg.
Granacher 2006	Decelerating perturbation while walking on a treadmill	Angular velocity of the ankle and knee joint, reflex activity (decelerating perturbation impulses), muscle onset latencies of TA, PE, and SO	Group 2 showed a decrease in onset latency, an enhanced reflex activity in the prime mover, and a decrease in maximal angular velocity of the ankle joint complex. No significant changes in groups 1 and 3.
Granacher 2009	ML perturbation impulse of a swinging platform while standing	Summed oscillations of the swinging platform in AP and ML directions, averaged EMG signals of TA and PE	Neither group showed any significant improvements.
Hamed 2018	Simulated forward falls (lean-and-release)	Limits of stability, margin of stability at release and TD, BOS at TD, duration from release until TD, rate	Both exercise groups, but not group 3, showed improvements in general.

		of increase in BOS, maximum voluntary isometric knee extension and ankle plantarflexion moment	
Hatzitaki 2009	Avoiding pendulum-like obstacle moving toward the participants' face in the sagittal plane without lifting their feet while standing on a platform	Peak of COP amplitude (APA and response phase), time to peak COP (APA and response phase), maximum trunk roll velocity, onset time of the APA	Group 1 showed significantly reduced COP response amplitude and increased maximum trunk roll velocity. APA onset time was significantly smaller for both Group 1 and 2.
Hu 1994	Horizontal platform translations while standing	Frequency of onset of muscles (GA, hamstrings, TA, quadriceps, trunk extensor, trunk flexor, neck extensor, neck flexor), muscle onset latencies, sequence of muscle onsets, averaged integrated EMG amplitude, joint angle patterns	Group 1 showed decreased onset frequency of the antagonist leg muscles, shortened onset latency of the neck flexor muscle, decreased response frequency of antagonist muscles, increased response frequency of the trunk flexor muscles, and decreased maximal excursion of the first trial of the ankle joint rotation versus group 2.
Inacio 2018	Stepping induced by lateral waist-pulls to the side of the limb where the weight was laterally transferred initially (50%, 65% and 80% BW)	Incidence of stabilizing single lateral recovery steps, lift-off time of the stepping foot, downward COM momentum at step lift-off, net hip abduction torque and power during the pre-step weight transfer phase, muscle activation of TFL, Gmed, and ADD	Group 1 showed a significantly increased incidence of stabilizing single lateral steps at 80% body mass pre-load, reduced step lift-off time at 50% body mass, and decreased downward momentum of the body COM at 80% body mass. In addition, group 1 showed increased hip abductor net joint torque, power, and abductor-adductor rate of neuromuscular activation.
Jagdhane 2016	Pendulum impact applied to the shoulders while standing	APA muscle activities or MG, TA, BF, RF, EO	Group 1, but not group 2, showed early onsets of APA activity prior to the external perturbations.
Kim 2010	Laboratory-induced slip while walking	Heel contact velocity, COM velocity, transitional acceleration of the whole body COM, step length, required coefficient of friction (friction demand), slip severity	Decreases in heel contact velocities and the friction demand characteristics and increase in transitional acceleration of the whole body COM in group 1 and 2. No intergroup differences in COM velocity, step length, and slip severity.
Klamroth 2019	Mini-BESTest	Reactive postural balance section	Group 1 showed a greater number of subjects with an improvement in reactive balance versus

			group 2.
Lacroix 2016	(1) Treadmill perturbation in the transverse plane while standing (2) Clinical push and release test	(1) summed oscillations of the platform in ML and AP directions; and (2) the number of steps and quality of the recovery	Group 1 and 2 showed improvements in the clinical push and release test. No between-group differences in the ability to compensate following platform translations.
Li 2009	Surface tilt perturbation of 18° generating ankle inversion while standing	Muscle onset latencies of RF, ST, gastrocnemius, and TA	Group 1 showed a significant decrease in ST muscle latency versus group2. No between-group differences in other muscles.
Ma 2019	Posterior-to-anterior trunk perturbation	Muscle onset latencies of MH and gastrocnemius, COP path length, and velocity	The muscle onset latency of gastrocnemius was longer in Group 1 versus Group 2. No between-group differences in other outcomes.
Mansfield 2010	Surface translation and/or cable pull (pelvic level): (1) stepping evoked by forward and backward perturbations while standing, (2) stepping evoked by leftward and rightward perturbations while walking in place, (3) grasping evoked by backward perturbations while standing	**All stepping reactions**: frequency of multi-step reactions, **AP stepping reactions**: frequency of extra lateral steps, frequency of reactions with more than two AP steps, foot-off time, foot-contact time, **ML stepping reactions**: frequency of foot collisions, crossover steps, **Grasping reactions**: handrail contact time, biceps muscle onset latency, frequency of grasping errors, **Forward fall stepping reactions**: forward step displacement, lateral step displacement, **Backward fall stepping reactions**: backward step displacement, lateral step displacement.	Group 1 showed greater reductions in the frequency of multi-step reactions and foot collisions during surface translations, but not cable pulls. Group 1 showed greater reductions in handrail contact time versus group 2 for cable pulls.
Marigold 2005	Platform translations (forward and backward directions) while standing	Muscle onset latencies of TA and RF for the forward translations and MG and BF for the backward translations, number of falls during the platform translations	Group 1 showed greater improvements in step reaction time, paretic RF postural reflex onset latency, and the number of induced falls versus group 2.
Morat 2019	Pendular movement of the platform in ML direction while standing	Total postural sway	Group 1 showed an improvement in the total postural sway.
Ni 2014	Dynamic posturography (EO and EC)	DMA score, time on the test, linear and angular displacements in the ML, AP, and up/down directions	Group 2 showed higher DMA scores and shorter time on the test versus group 1.
Ochi 2015	Simulated forward falls (lean-and-release)	spatiotemporal parameters (lift-off time, step time, step length, step velocity, trunk angle at initial lean and foot contact), EMG onset times, timing of first-	Both groups showed extended step length and increased peak EMG of knee flexor and extensor muscles. Group 1 showed increased

		peak EMG amplitude, and normalized peak EMG amplitude of RF, VL, BF, TA, LG	step velocity and peak EMG of the plantar flexors.
Okubo 2019	Laboratory-induced slip or trip while walking	Rate of falls, margin of stability, XCOM position, step length, step height, trunk sway range, slip speed, slip distance	Group 1 showed a lower rate of falls versus group 2. During a trip, group 1's XCoM position was less anterior, the recovery stepping foot was higher, and the trunk sway range was smaller versus group 2. During a slip, group 1 had less posterior XCoM position, shorter backward step length, and smaller trunk sway range versus group 2.
Pamukoff 2014	Simulated forward and lateral falls (lean-and-release)	The largest angle from which the participant could successfully recover their balance	No between-group differences in all outcomes.
Parijat 2012	Laboratory-induced slip while walking	Incidence of falls, slip severity (slip distance and peek sliding heel velocity), joint angles (ankle, knee, hip, and trunk angles at HC, peak angles of ankle, knee, hip, and trunk), peak joint angular velocity (ankle, knee, hip, trunk), muscle activation onset and time to peak activations of MG, TA, MH, and VL, coactivations (peak ankle and knee co-activities, time to peak ankle and knee co-activities), non-slipping foot response time (toe-off, foot-onset, foot down, unperturbed foot reaction time), unperturbed foot reaction time	Group 1 showed greater reductions in the incidence of falls and slip severity (slip distance and peak sliding heel velocity) versus group 2. Group 1 showed proactive adjustments (increased COM velocity and transitional acceleration), and reactive adjustments (reduction in muscle onset and time to peak activations of knee flexors and ankle plantar flexors, reduced ankle and knee coactivation, reduced slip displacement, and reduced time to peak knee flexion, trunk flexion, and hip flexion velocities). Group 1 showed a shorter reaction time of the unperturbed foot versus group 2.
Parijat 2015a	Laboratory-induced slip while walking	Incidence of falls during testing, joint angles (ankle, knee, hip, and trunk angles at HC, peak angles of ankle, knee, hip, and trunk), peak joint angular velocity (ankle, knee, hip, trunk), muscle activation onset and time to peak activations of MG, TA, MH, and VL, coactivations (peak ankle and knee co-activities, time to peak ankle and knee co-activities).	Group 1 showed proactive adjustments (increased trunk flexion at heel contact) and reactive adjustments (reduced time to peak activations of knee flexors, reduced knee coactivation, reduced time to trunk flexion, and reduced trunk angular velocity).
Parijat 2015b	Laboratory-induced slip while walking	Incidence of falls during testing, slip distance, peak sliding heel velocity	Group 1 showed a reduced incidence of falls, slip distance, and peak sliding heel velocity.
Pluchino	Dynamic posturography	DMA score, translational movements (AP, ML,	No significant group differences in all

2012		up/down), rotational movements (flexion/extension, lateral flexion, core rotational)	outcomes.
Qutubuddin 2007	Dynamic posturography	Adaptation test scores	No significant group differences in all outcomes.
Rieger 2020	Treadmill perturbation in AP and ML directions while walking	Deviations of perturbed gait trunk velocity from unperturbed gait	Both groups showed improvements in AP and ML directions, but no group differences were reported.
Rossi 2014	Platform translations in forward and backward directions while standing	EMG amplitude of RF, VMO, ST, TA, MG, and SO in the early (0-200 ms), intermediate (201-400 ms), and late (401-600 ms) phases	Greater amplitude for group 1 than for group 2 after training for the TA, MG, and SO muscles at the early phase and for the SO muscle at the intermediate phase. No difference in the late phase.
Santos 2017	BESTest	Reactive postural responses section	No significant group difference.
Schlenstedt 2015	Platform translations in forward and backward directions while standing	COM displacement	No significant group difference.
Shimada 2003	Manual perturbation test (shoulder was pulled backwards)	Responses were scored (0-2)	No significant group difference.
Sohn 2015	Laboratory-induced slip while walking	COP area and distance, fall frequency	Group 1 and 2 showed improvements in all outcomes in comparison to group 3.
Thomas 2016	Platform translations in ML direction while standing (tandem stand and one-leg stand)	Time of standing on the moving platform without holding to the handrail, accumulated accelerations	Both groups showed improvements in the time of standing and accumulated accelerations. No group differences were reported.
Wang 2019	Laboratory-induced slip while walking	Slip recovery classification (fall, backward loss of balance, or full recovery), dynamic stability control (proactive stability control at slipping foot TD and reactive stability control at recovery foot lift off)	Group 1 showed fewer falls and greater proactive and reactive stability versus group 2.
Wolf 1997	Angular perturbation (toes up and toes down) of a platform while standing on the Chattexc Balance System	Dispersion measures, measures of center of balance in X and Y axes	Dispersion under toes up and down conditions were reduced substantially in group 1 versus group 2 and 3. Center of balance in X axis under toes up condition showed a greater decrease in group 1 versus group 2 and 3. Center of balance in Y axis increased in group 3.

Wooten 2018	Dynamic posturography	DMA score, total time on the test	No significant group differences.

COM, center of mass; XCOM, extrapolated center of mass; COP, center of pressure; TD, touch down; HC, heel contact; BOS, base of support; EMG, electromyograph; TA, tibialis anterior; MG, medial gastrocnemius; LG, lateral gastrocnemius; SO, soleus; PE, peroneus; RF, rectus femoris; VL, vastus lateralis; VMO, vastus medialis oblique; BF, biceps femoris; MH, medial hamstring; ST, semitendinosus; TFL, tensor fascia latae; Gmed, gluteus medius; ADD, adductor magnus; RA, rectus abdominis; EO, external oblique; ES, erector spinae; AP, anteroposterior; ML, mediolateral; APA, anticipatory postural adjustment; EO, eyes open; EC, eyes closed; DMA, Dynamic motion analysis; BW, body weight.

Supplementary Table 3. Summary table of the reviewers' judgements for the risk of bias of each study

Study	Randomization process	Deviations from the intended interventions	Missing outcome data	Measurement of the outcome	Selection of the reported result	Overall
Allin 2020	Some concerns	Low	Low	Low	Some concerns	Some concerns
Arampatzis 2011	Some concerns	Low	High	Low	Some concerns	High
Arghavani 2020	Some concerns	Low	High	Low	Some concerns	High
Beling 2009	Some concerns	Low	Low	Low	Some concerns	Some concerns
Bieryla 2007	Some concerns	High	Low	Low	Some concerns	High
Bogaerts 2007	Some concerns	Low	High	Low	Some concerns	High
Cabrera-Martos 2020	Low	Low	Low	Low	Low	Low
Cherup 2019	Some concerns	Low	High	Low	Some concerns	High
Chyu 2010	Low	Some concerns	Low	Low	Some concerns	Some concerns
Donath 2016	Low	Some concerns	High	Low	Some concerns	High
Gatts 2007	Some concerns	Some concerns	High	Low	Some concerns	High
Gatts 2008	Some concerns	Some concerns	High	Low	Some concerns	High
Granacher 2006	Some concerns	Low	Low	Low	Some concerns	Some concerns
Granacher 2009	Some concerns	Low	Low	Low	Some concerns	Some concerns
Hamed 2018	Low	Some concerns	Low	Low	Some concerns	Some concerns
Hatzitaki 2009	Some concerns	Some concerns	Low	Low	Some concerns	Some concerns
Hu 1994	Some concerns	Some concerns	Low	Low	Some concerns	Some concerns
Inacio 2018	Some concerns	Some concerns	Low	Low	Some concerns	Some concerns
Jagdhane 2016	Some concerns	Some concerns	Low	Low	Some concerns	Some concerns
Kim 2010	Some concerns	Some concerns	Low	Low	Some concerns	Some concerns
Klamroth 2019	Some concerns	Some concerns	High	Low	High	High
Lacroix 2016	Low	Some	High	Low	Some	High

		concerns			concerns	
Li 2009	Some concerns	Some concerns	High	Low	Some concerns	High
Ma 2019	Low	Some concerns	Low	Low	Low	Some concerns
Mansfield 2010	Low	Some concerns	High	Low	Low	High
Marigold 2005	Low	Low	High	Low	Some concerns	High
Morat 2019	Some concerns	Low	High	Low	Some concerns	High
Ni 2014	Some concerns	Some concerns	High	Low	Some concerns	High
Ochi 2015	Some concerns	Low	Low	Low	Some concerns	Some concerns
Okubo 2019	Low	Some concerns	Low	Low	Low	Some concerns
Pamukoff 2014	Some concerns	Low	High	Low	Some concerns	High
Parijat 2012	Some concerns	Some concerns	Low	Low	Some concerns	Some concerns
Parijat 2015a	Some concerns	Some concerns	Low	Low	Some concerns	Some concerns
Parijat 2015b	Some concerns	Some concerns	Low	Low	Some concerns	Some concerns
Pluchino 2012	Low	Low	High	Low	Some concerns	High
Qutubuddin 2007	Some concerns	Some concerns	High	Low	Some concerns	High
Rieger 2020	Some concerns	Some concerns	Low	Low	Some concerns	Some concerns
Rossi 2014	Some concerns	Some concerns	Low	Low	Low	Some concerns
Santos 2017	Low	Low	High	Low	Low	High
Schlenstedt 2015	Some concerns	Some concerns	High	Low	Low	High
Shimada 2003	Some concerns	Some concerns	Low	Low	Some concerns	Some concerns
Sohn 2015	Some concerns	Some concerns	Low	Low	Some concerns	Some concerns
Thomas 2016	Some concerns	Some concerns	Low	Low	Some concerns	Some concerns
Wang 2019	Some concerns	Low	Low	Low	Low	Some concerns
Wolf 1997	High	Some concerns	High	Low	Some concerns	High
Wooten 2018	Some concerns	Low	High	Low	Some concerns	High
